# Development and evaluation of an eHealth self-management intervention for patients with chronic kidney disease in China: protocol for a mixed-method hybrid type 2 trial

**DOI:** 10.1186/s12882-020-02160-6

**Published:** 2020-11-19

**Authors:** Hongxia Shen, Rianne van der Kleij, Paul J. M. van der Boog, Xiaoyue Song, Wenjiao Wang, Tongtong Zhang, Zhengyan Li, Xiaoping Lou, Niels Chavannes

**Affiliations:** 1 Department of Public Health and Primary Care, Leiden University Medical Centre, Leiden, Netherlands; 2 Department of Nursing, The First Affiliated Hospital of Zhengzhou University, Zhengzhou University, Zhengzhou, China; 3 Department of Obstetrics and Gynaecology, Erasmus Medical Center, Rotterdam, Netherlands; 4 Department of Nephrology, Leiden University Medical Centre, Leiden, Netherlands; 5 School of Nursing and Health, Zhengzhou University, Zhengzhou, China; 6 Department of Nephrology, The First Affiliated Hospital of Zhengzhou University, Zhengzhou University, Zhengzhou, China

**Keywords:** eHealth, Self-management, Chronic kidney disease, China, Hybrid design, Implementation

## Abstract

**Background:**

Chronic kidney disease (CKD) is a significant public health concern. In patients with CKD, interventions that support disease self-management have shown to improve health status and quality of life. At the moment, the use of electronic health (eHealth) technology in self-management interventions is becoming more and more popular. Evidence suggests that eHealth-based self-management interventions can improve health-related outcomes of patients with CKD. However, knowledge of the implementation and effectiveness of such interventions in general, and in China in specific, is still limited. This study protocol aims to develop and tailor the evidence-based Dutch ‘Medical Dashboard’ eHealth self-management intervention for patients suffering from CKD in China and evaluate its implementation process and effectiveness.

**Methods:**

To develop and tailor a Medical Dashboard intervention for the Chinese context, we will use an Intervention Mapping (IM) approach. A literature review and mixed-method study will first be conducted to examine the needs, beliefs, perceptions of patients with CKD and care providers towards disease (self-management) and eHealth (self-management) interventions (IM step 1). Based on the results of step 1, we will specify outcomes, performance objectives, and determinants, select theory-based methods and practical strategies. Knowledge obtained from prior results and insights from stakeholders will be combined to tailor the core interventions components of the ‘Medical Dashboard’ self-management intervention to the Chinese context (IM step 2–5). Then, an intervention and implementation plan will be developed. Finally, a 9-month hybrid type 2 trial design will be employed to investigate the effectiveness of the intervention using a cluster randomized controlled trial with two parallel arms, and the implementation integrity (fidelity) and determinants of implementation (IM step 6).

**Discussion:**

Our study will result in the delivery of a culturally tailored, standardized eHealth self-management intervention for patients with CKD in China, which has the potential to optimize patients’ self-management skills and improve health status and quality of life. Moreover, it will inform future research on the tailoring and translation of evidence-based eHealth self-management interventions in various contexts.

**Trial registration:**

Clinicaltrials.gov NCT04212923; Registered December 30, 2019.

**Supplementary Information:**

The online version contains supplementary material available at 10.1186/s12882-020-02160-6.

## Background

### Prevalence and burden of chronic kidney disease

Chronic kidney disease (CKD) poses a significant threat to public health [1–3]. Globally, more than 70 million individuals are affected by CKD [4]. In China, an estimated 10.8% (119.5 million) of adults suffer from CKD [5]. CKD is defined as abnormalities of kidney structure or function, present for more than 3 months, with severe implications for health [6]. CKD is chronic and categorized into five stages based on the level of glomerular filtration rate (GFR) and albuminuria [6]. Numerous detrimental health outcomes are linked to CKD [7]. Also, CKD increases mortality risk and hospitalization rates, and negatively impacts the quality of life [7–9]. Additionally, health-related and societal costs of CKD are considerable and constitute a substantial economic burden [10–12].

### Self-management and eHealth interventions for CKD

Interventions that support disease self-management (further referred to as ‘self-management interventions’) can have a significant impact on the health and quality of life of patients suffering from chronic conditions in general [13], and patients with CKD in specific [14–16]. Self-management support is often defined as “*[……] improving chronic illness outcomes consisting of patient-centered attributes (involving patients as partners; [……]), provider attributes (possessing adequate knowledge, skills, attitudes in providing care), and organizational attributes (putting an organized system of care in place, having multidisciplinary team approach, using tangible and social support)*” [17].

In the last decade, the use of electronic health (eHealth) technology in self-management interventions has become more and more popular. EHealth technology can facilitate remote patient-provider communication and exchange of (health) data and has the potential to increase healthcare accessibility and efficiency [18]. EHealth-based self-management interventions have been shown to improve health-related outcomes, such as blood pressure (BP) control and medication adherence [19, 20], and found to be feasible and acceptable for patients with CKD and care professionals [19]. Hence, the use of eHealth self-management interventions for patients with CKD has become increasingly popular. Knowledge of the implementation and effectivity of such interventions in China and other developing countries is, however, still lacking [21].

### Medical dashboard

Researchers from the Leiden University Medical Center (LUMC) developed ‘Medical Dashboard’, an eHealth intervention to help support and involve patients with CKD in their disease self-management. This platform is used in the Outpatient Clinic Kidney Transplant of the LUMC since February 2016. Via Medical Dashboard, patients can monitor their health from home (e.g., BP, weight), and can exchange health data with their care professionals. Moreover, during consultations in the outpatient clinic, care professionals and patients can also use Medical Dashboard to set personal health goals such as BP control and nutrition management (e.g., energy). In a randomized controlled trial (RCT), the use of “Medical Dashboard” has been shown to improve patients’ adherence to sodium restriction intake and BP control [14]. Also, patients reported being highly satisfied with the online disease management system used in the platform [22]. All core intervention components of ‘Medical Dashboard’ and their supporting evidence base are presented in Additional file 1.

### Opportunities for eHealth interventions in China

There is significant support and momentum for the implementation of eHealth based self-management interventions in China. China had 731 million internet users (penetration rate 53%) and 1.3 billion mobile phone users (penetration rate of 90%) in 2016, and this number is still growing [23–26]. Furthermore, policymakers and care experts in China have recently launched the national health strategy ‘Healthy China 2030’. This strategy describes eHealth technology as an essential pillar to improve disease self-management as well as the accessibility and cost-effectiveness of care in rural areas. Moreover, it views eHealth technology as the preferred medium to reach one of the main goals: ‘*enable everyone to be involved in health, share health, and be responsible for health*’ [27, 28]. Also, the prevalence rate and severe adverse health outcomes of CKD have put it high on the public health agenda in China.

### Study aims and research methods

In conclusion, eHealth self-management interventions have the potential to fundamentally improve the quality of life and health outcomes of patients suffering from CKD in China. The Medical Dashboard based self-management intervention has been researched extensively and proven effective. Also, our research team has a close relationship with its developers and is therefore able to amend and upscale the intervention globally. Therefore, we aim to tailor the evidence-based Dutch intervention ‘Medical Dashboard’ to the Chinese context and evaluate its implementation process and effectiveness. To this end, we will use an intervention mapping (IM) approach comprising six steps: (1) a needs assessment, (2) preparation of change objectives matrices, (3) selection of theory-informed intervention methods and strategies, (4) development of a tailored ‘Medical Dashboard’ based intervention plan, (5) development of an implementation - and (6) evaluation plan.

In correspondence with the steps of IM [29], we aim to:

- ➢ *Phase 1: Needs, beliefs and perceptions (Step 1 of IM)*

Examine the needs, beliefs, perceptions of patients with CKD and care providers towards disease self-management and eHealth interventions;

- ➢ *Phase 2: Intervention and implementation development & planning (Step 2–5 of IM)*

Tailor the core components of the ‘Medical Dashboard’ self-management intervention for patients with CKD to the Chinese context;

- ➢ *Phase 3: Intervention evaluation (Step 6 of IM)*

Employ a hybrid type 2 trial to:

- Evaluate the effectiveness of the intervention using a cluster RCT with two parallel arms;
- Evaluate implementation integrity (fidelity) and determinants of implementation.

## Methods

The study has been approved by the Ethics Committee of the First Affiliated Hospital of Zhengzhou University (reference number 2019-KY-52).

### Study setting

All study phases are (to be) conducted in the First Affiliated Hospital of Zhengzhou University in the Henan province in China. Henan is one of the biggest provinces of China, and it accounts for 9% of the rural Chinese population. An estimated 16.4% (12 million) of adults suffer from CKD in rural areas in Henan [30]. The Department of Nephrology of the First Affiliated Hospital of Zhengzhou University has five sub-units with approximately 276 beds; more than 60,000 patients with CKD visit the Outpatient Clinic of Department of Nephrology each year.

### Overview of study design

An overview of the study flow following the six steps of IM is displayed in Table 1.

**Table 1:** Overview of study phases

Phase	IM steps	Activities
I	Step 1 Conduct needs assessment	• Establish an intervention monitoring group • Perform a systematic literature review • Conduct a mixed-methods study into needs, beliefs & perceptions of patients with chronic kidney disease and care providers toward chronic kidney disease (self-management) and the use of eHealth (self-management) interventions
II	Step 2 Identify outcomes, performance objectives, and determinants	• Formulate program outcomes • Specify performance objectives • Specify determinants of change • Map the performance objectives to the determinants and create a matrix of change objectives
	Step 3 Select theory-based methods and practical strategies	• Review potentially relevant theoretical methods • Match each determinant to the relevant method(s) • Translate methods into practical strategies to target each determinant • Monitoring group reaches consensus on methods and practical strategies
	Step 4 Develop a tailored ‘Medical Dashboard’ based intervention (plan)	• Develop an intervention plan by tailoring the core components of the Dutch Medical Dashboard to the Chinese context • Member check with the target population
	Step 5 Develop an adoption- and implementation plan	• Identify potential adopters and implementers • Specify program use outcomes and performance objectives • Specify determinants of change • Map the performance objectives to the determinants and create a matrix of change objectives • Design a plan for adoption and implementation • Member check with the target population
III	Step 6 Develop an intervention evaluation plan	• Specify the two-arm, hybrid 2 trial design and: -Develop the effectiveness evaluation plan -Develop the implementation evaluation plan

### Phase 1

#### Aim

Preliminary evidence suggests that both patients’ and care providers’ needs, beliefs (i.e., an idea or principle judged to be true) and perceptions (i.e., the organized cognitive representations that individuals have about a subject) of disease (self-management) can influence their display of health behaviors and uptake of (self-management) interventions [31–34]. Therefore, following step 1 of IM, we will first conduct a needs assessment and examine the needs, beliefs, perceptions of patients with CKD and care providers towards disease (self-management) and the use of eHealth interventions.

#### Design

##### Intervention monitoring group

First, an intervention monitoring group including both Dutch and Chinese experts and other key stakeholders will be established. This group will consist of two researchers, one nephrologist, one nurse in CKD practice, one implementation specialist, one primary care clinician, one rehabilitation therapist, one patient with CKD, one patient advisor, and one informal caregiver. The expert group has ample experience with CKD care and the implementation of (eHealth) self-management interventions. The intervention monitoring group will meet monthly throughout all IM steps to discuss progress and the execution of major deliverables such as the needs assessment (e.g. program goals), intervention development (e.g. intervention content, delivery strategies), and evaluation planning (e.g. inclusion, outcome choice, analysis).

### Literature review

A scoping literature review will be conducted to identify relevant evidence on needs toward disease management of patients with CKD and care providers. The search strategy is already developed in collaboration with a certified librarian (see Additional file 2).

### Mixed-method study

#### Research methodology

**Fig. 1:**
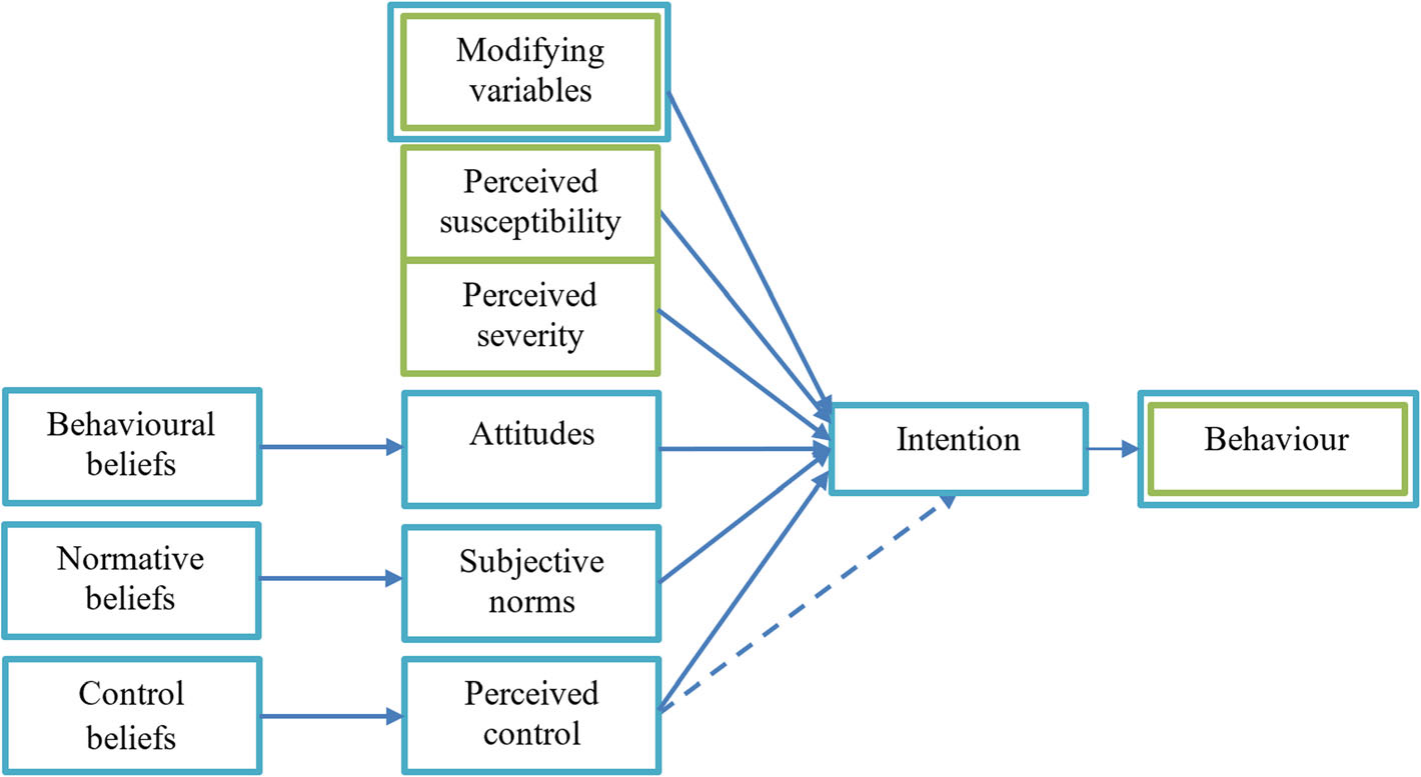
Adapted version of the theoretical framework of Brakema et al (submitted). A combination of concepts of the Health Beliefs Model (green) and the Theory of Planned Behavior (blue)

We will conduct a mixed-method study to gain insight into the needs, beliefs, perceptions of patients with CKD, and care providers towards disease (self-management) and the use of eHealth (self-management) interventions. This study will include face to face interviews, focus group discussions, observations, and survey research. Methods will build on an adapted version of the theoretical framework on beliefs and perceptions towards chronic lung disease used in FRESH AIR (Brakema et al., submitted). This adapted framework combines the Health Belief Model [35] and the Theory of Planned Behavior [36] and focuses on individuals’ beliefs and perceptions as well as the sociocultural context in which the individual resides (see Fig. 1).

We will explore patients’ and care providers’: (1) beliefs and perceptions towards CKD and disease self-management, (2) needs towards CKD self-management, and (3) needs, beliefs, perceptions towards the use of eHealth interventions in disease self-management. The survey will consist of three validated measures: (1) ‘The Brief Illness Perception Questionnaire’ (BIPQ) [37], (2) ‘Chronic Kidney Disease Self-management instrument’ (CKD-SM) [38], and (3) ‘Chinese eHealth Literacy Scale’ (C-eHEALS) [39]. Each questionnaire will be tested on usability, feasibility, and acceptability by ten volunteers patients before they are to be used on a larger scale. If any issues arise, the questionnaires will be adapted accordingly, for instance, by reformulating specific questions.

#### Sample size calculation

For the qualitative part, following principles of “purposive and convenience sampling” [40], the inclusion of participants will be based on opportunity, willingness to participate, and creation of diversity (e.g., different stages of CKD, age, gender) in our sample. We will also use snowball sampling [41], in which participants will be asked if they know any other individuals who could participate in the study. As there are no defined rules for calculating sample size in qualitative studies [42], target numbers are set for the data collection based on previous literature and our experience in previous studies (Table 2). The definitive sample size for all qualitative research elements will be determined based on when data saturation is achieved, which is the point when no new or relevant information is identified through the preliminary analysis of the data [43]. For the quantitative part, as a rule of thumb, the sample size should be 5–10 times the number of items in the questionnaires [44]. Therefore, we aim to recruit at least 230 patients in the quantitative survey (Table 2).

**Table 2:** Sample size calculation in a mixed-method study

Method	Sampling	Participants	Sample (range between records)
Face to face interview	Purposive, Convenience	• Care providers • Patients	10–15 care providers minimum 10–15 patients minimum
Focus group discussion	Purposive, Convenience	• Patients	2–3 groups of 8–10 patients in total
Observation	Purposive, Convenience	• Care providers • Patients	10–15 observations minimum
Survey	Randomly	• Patients	230 patients minimum

#### Study population

The eligibility criteria of participants are detailed in Table 3. Approximately 200 care providers, of which 60 are nephrologists, in the Department of Nephrology of the First Affiliated Hospital of Zhengzhou University are available for potential recruitment. The methods to be used differ between patients and care providers following the relevant group- and context characteristics (see details in Table 4). For instance, focus groups cannot be held with care providers as they (1) cannot be of duty all at the same time, and (2) work with a tight schedule, and finding a time slot that suits all care providers is very difficult. Moreover, we feel that patients with CKD would be comfortable discussing their needs towards eHealth self-management interventions in a focus group setting, but not their needs and beliefs towards their disease in general. Hence, we will plan to discuss this topic in face-to-face interviews. More details on the methods use and relevant research materials used are presented in Additional file 3.

**Table 3:** Eligibility criteria for patients with chronic kidney disease and care providers

Category	Participant eligibility criteria
Inclusion criteria	• Patients: (1) aged over 18 years old; (2) a diagnosis of chronic kidney disease (CKD) with markers of kidney damage or a glomerular filtration rate of less than 60 ml/min/1.73m^2^ persisting for ≥3 months based on Kidney Disease Improving Global Outcomes (KDIGO) guidelines [6]; (3) all CKD stages (stage 1–5) following the KDIGO staging of CKD [6]; (4) Chinese speaking. • Health care providers (1) who work in the Department of Nephrology of the First Affiliated Hospital of Zhengzhou University (2) are able to implement the intervention in their daily practice
Exclusion criteria	• Individuals unable to provide written informed consent and use electronic application due to physical disabilities such as eyesight problems or mental disabilities such as psychosis, personality disorders or schizophrenia (final decision for exclusion will be made by the treating physician) • Individuals unable to write or read.

**Table 4:** Field methods used for topics

Method	Care providers			Patients		
	Beliefs, perceptions, toward chronic kidney disease and self-management	Needs toward chronic kidney disease self-management	Needs, beliefs, perceptions toward eHealth self-management interventions	Beliefs, perceptions toward chronic kidney disease and self-management	Needs toward chronic kidney disease self-management	Needs, beliefs, perceptions toward eHealth self-management interventions
Face to face interview	X	X	X	X	X	X
Focus group discussion						X
Observation	X	X	X	X	X	X
Survey				X	X	X

### Phase 2

#### Aim

Following step 2–5 of IM, we aim to tailor the core interventions components of the ‘Medical Dashboard’ self-management intervention to the Chinese context following the results of the needs assessment performed in Phase I.

#### Design

**Fig. 2:**
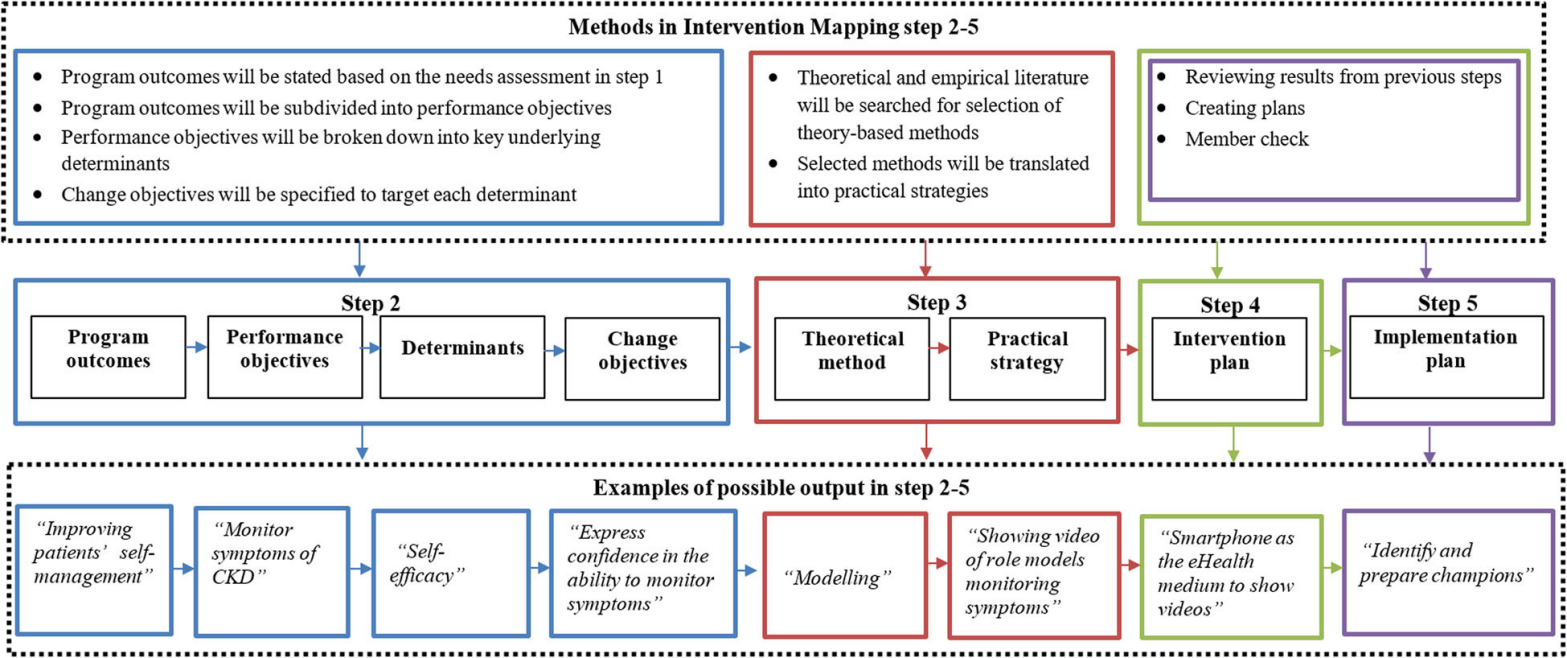
Methods and examples of the possible output of Intervention Mapping step 2–5. Step 2 (blue), step 3(red), step 4(green), step 5(purple)

All the IM concepts used in the steps below are operationalized and further detailed in Table 5 and Fig. 2.

**Table 5:** The concepts from Intervention Mapping step 2–5

Concept of Intervention Mapping	Definition in Bartholomew LK et al. [29]
**Step 2**	
Program outcome	Desired changes in the behavior and the environmental conditions
Performance objective	The required actions to accomplish the change in the behavioral and environmental outcomes
Determinant	Factors that are associated with the performance of behavior
Change objective	Specific goals stating what should change at the determinants for program outcomes in different level
**Step 3**	
Theoretical method	General technique or process for influencing changes in the determinants of behaviors and environmental conditions
Practical strategy	A specific technique for the practical use of theoretical methods in ways that fit with the target group and the context in which the intervention will be conducted
**Step 4**	
Intervention plan	A plan detailing intervention scope and sequence including delivery channels, themes, and list of the intervention materials needed
**Step 5**	
Implementation plan	A plan detailing how intervention adoption and implementation can be supported and maintained over time.

### Step 2: preparing matrices of change objectives

First, we will formulate *program outcomes* [29] on all levels as defined in the socio-ecological model [45]. This model will help us to understand the complex interplay between individual, interpersonal, community, and societal outcomes. Second, we will subdivide program outcomes into *performance objectives* [29]. Third, as each performance objective can only be reached if matching behavioral determinants are addressed, we will break each performance objective down into *key underlying determinants* [29]. We will use the Theoretical Domains Framework (TDF) to support the identification and selection of relevant determinants of behavior [46]. Two researchers will independently identify the determinants, and discrepancies will be resolved through discussions. Also, the intervention monitoring group will evaluate the determinants selected based on relevance and changeability, using the four possible consensus-based recommendation levels proposed by Michie et al. [46]. Finally, based on the determinants identified, we will specify *change objectives* [29].

### Step 3: selecting theory-informed intervention methods and practical strategies

We will first review the literature and identify relevant *theoretical methods* that can potentially induce a change in the determinants identified in step 2 [29]. Second, we will match the selected methods with specific change objectives. Third, the selected methods will be translated into practical strategies to target each determinant. Finally, the intervention monitoring group will rank the practical strategies per method [46] and ensure that these methods and practical strategies match with the program goals.

### Step 4: develop a tailored ‘medical dashboard’ based intervention (plan)

First, we will review the results of the needs assessment, the initial program’s logic model of change, and discuss intervention objectives, theoretical methods, and practical strategies for each level (e.g., individual, organization) specified in step 1–3. Second, the intervention monitoring group will have a meeting to amend, and if necessary, adapt the Medical Dashboard intervention. Only surface level adaptations will be made [47], such as the tailoring of educational content based on the results of the needs assessment, or by extending the intervention delivery medium to tablets or personal computers (listed in Additional file 1). To ensure the effectiveness of the Medical Dashboard based self-management intervention, we will not change the core self-management intervention components of Medical Dashboard that underline its effectivity, such as the provision of information support or self-monitoring. Also, the intervention monitoring group will create a plan for developing and testing the new version of the Medical Dashboard. Third, we will recruit five patients and five care providers to discuss the acceptability and feasibility of the intervention plan (member-check). To this end, we will use the ‘think aloud’ method [48], in which patients and care providers can speak aloud any words in their mind as they read through parts of the intervention plan. The think-aloud research method has been demonstrated to provide valid data on participant thinking and was successfully used in other intervention development studies [49, 50]. Based on the results obtained, further modifications will be made, resulting in a pre-tested version of the intervention plan ready for implementation in practice. The description of the intervention plan will follow the Template for Intervention Description and Replication [51].

### Step 5: develop an adoption and implementation plan

**Fig. 3:**
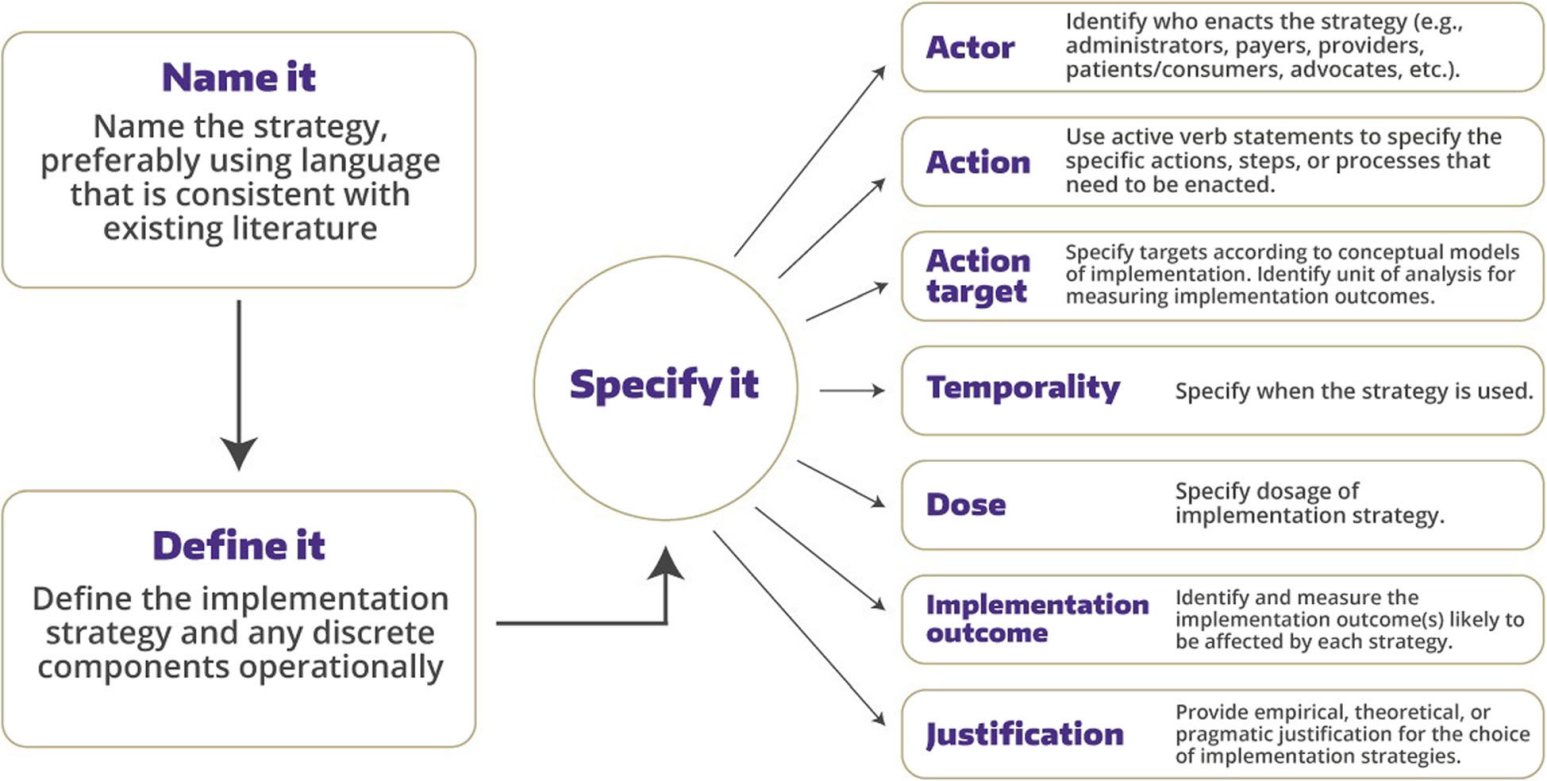
Guidance for specifying implementation strategies of Proctor EK et al. [52]

The goal of this step is to write a detailed adoption and implementation plan, containing relevant strategies to optimize intervention delivery and implementation (fidelity). First, we will discuss results obtained from step 1–4 and inventory local resources (e.g., connections with primary care clinics) that may facilitate intervention implementation. Second, based on all results obtained from previous steps and our previous systematic review [21], the intervention monitoring group will have a meeting to pragmatically identify potential adopters and implementers. Also, this group will demonstrate program use outcomes, performance objectives and related determinants of implementation. Third, the intervention monitoring group will design the implementation plan following Fig. 3 [52] based on Expert Recommendations for Implementing Change list of strategies [53]. Then, we will use the ‘think aloud’ method to obtain feedback from patients with CKD and care providers on the implementation plan. Finally, the adoption and implementation plan will be finalized with further modifications.

### Phase 3

#### Aim

Following step 6 of IM, we will establish an intervention evaluation plan. Our evaluation will follow a hybrid type 2 trial design, comprising of (1) a cluster RCT with two parallel arms to study effectiveness, and (2) a process evaluation to evaluate implementation integrity (fidelity) and determinants of implementation.

#### Design

**Fig. 4:**
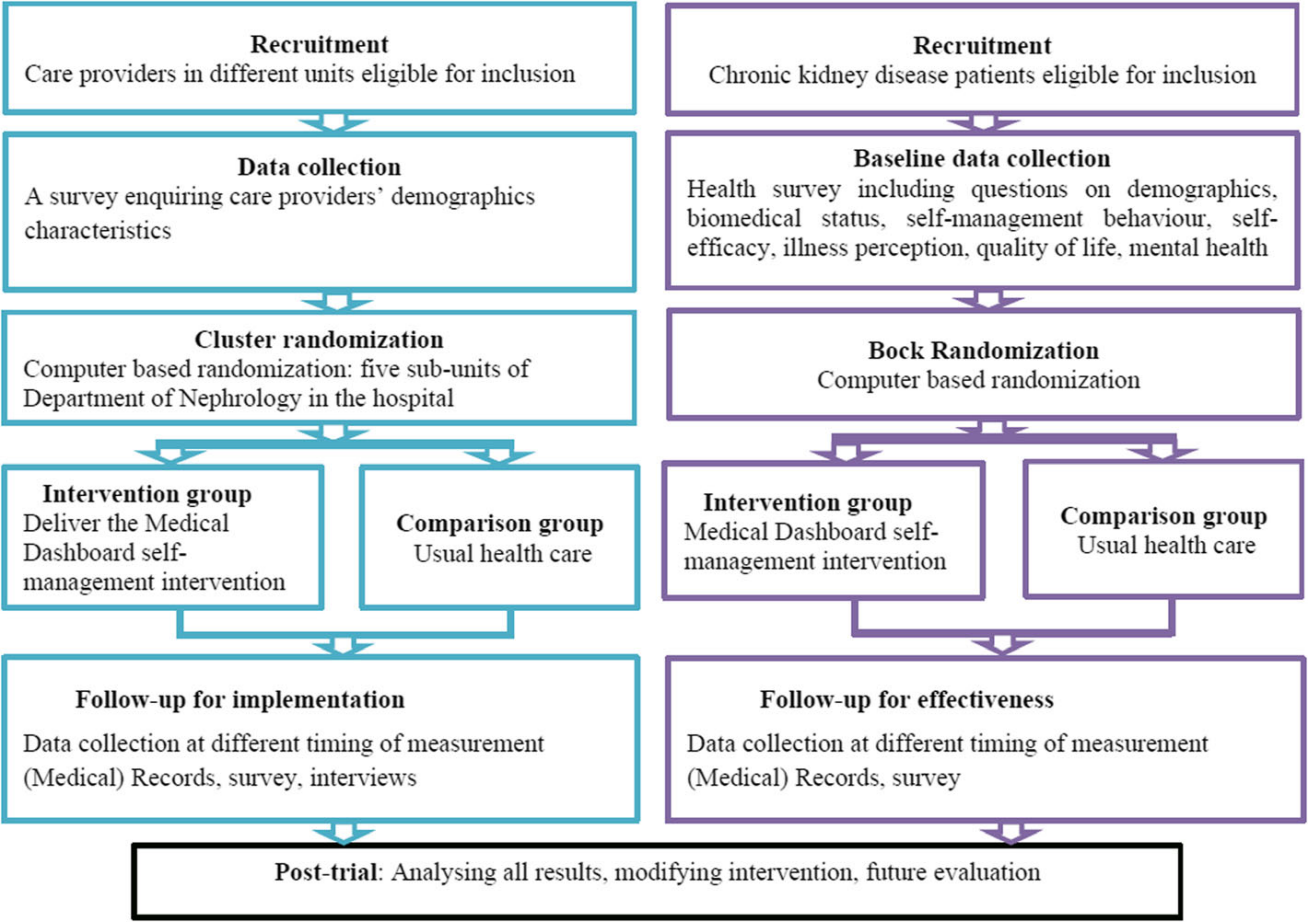
Study schema

This study will consist of a 9-month, cluster RCT with two parallel arms, integrated into a hybrid type 2 trial [54]. The trial design and corresponding study elements are detailed in Fig. 4. We selected an intervention duration of 9 months, as previous literature provides support that this intervention duration is sufficient to demonstrate the impact on several self-management outcome indicators [55, 56]. The Standard Protocol Items: Recommendations for Interventional Trials 2013 Statement is used to report the RCT protocol [57] (see Additional file 4), and the Standards for Reporting Implementation Studies will be followed for reporting the implementation study [58].

#### Intervention

Patients with CKD in the comparison group will receive usual care consisting of personalized in- and outpatient treatment based on symptoms experienced and disease severity, as outlined in the Kidney Disease Improving Global Outcomes (KDIGO) guidelines [6]. Patients with CKD in the intervention group will receive the usual care plus the culturally tailored ‘Medical Dashboard’ based self-management intervention for 9 months. Also, care providers in the intervention arm will implement the usual care plus the culturally tailored ‘Medical Dashboard’ based self-management intervention for 9 months. Those who are in the comparison group will implement the usual care. Before the start of the intervention, patients with CKD and care providers will receive a face-to-face training session on the use of Medical Dashboard. To avoid contamination, Medical Dashboard will only be made accessible for participants in the intervention group via a secure password-protected registration process.

#### Study population, recruitment & randomization

##### Effectiveness; RCT

**Fig. 5:**
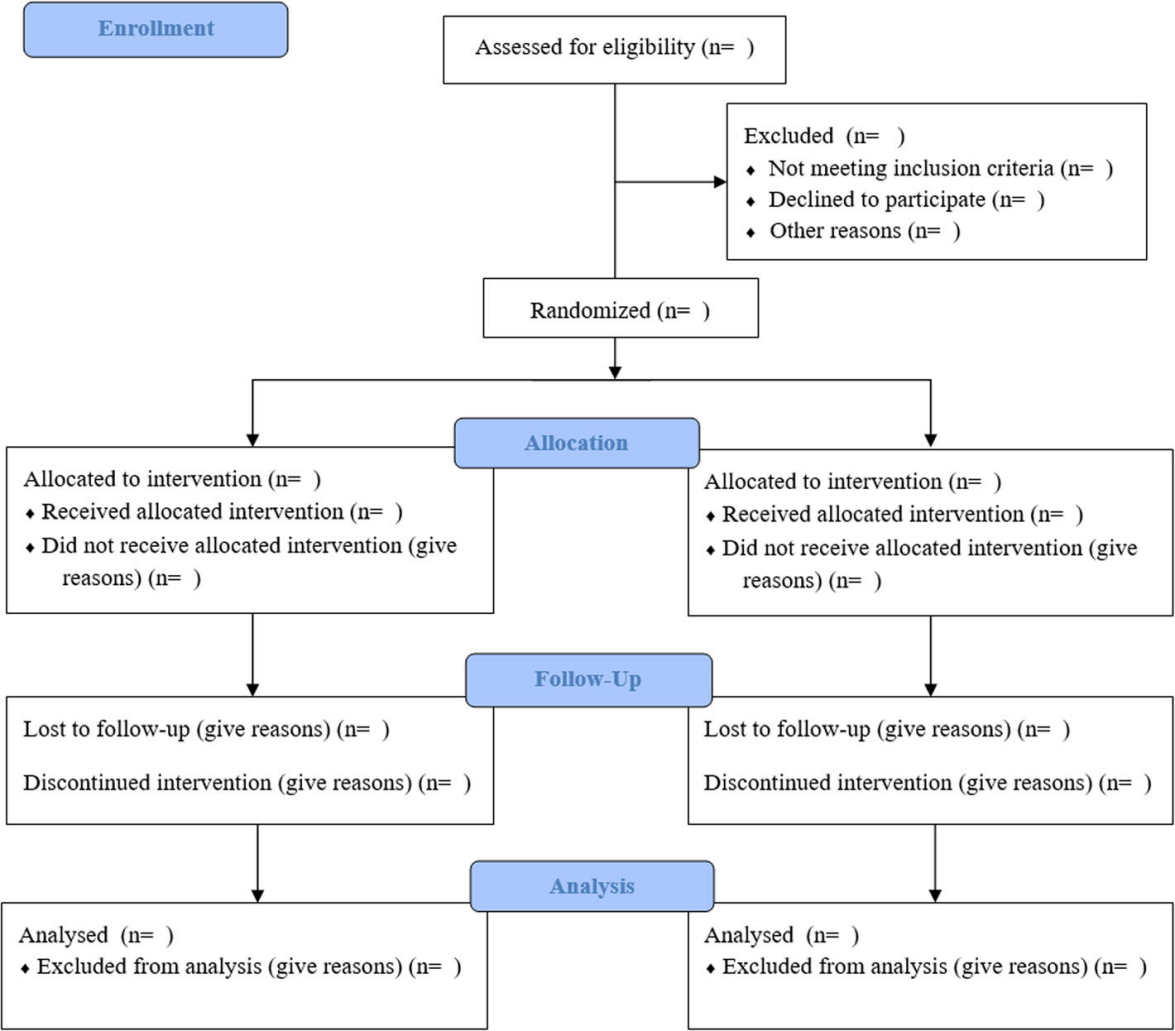
CONSORT flow diagram for our trial

Patients with CKD and care providers will be recruited from the First Affiliated Hospital of Zhengzhou University. Recruitment strategies, inclusion, and exclusion criteria are identical to those in phase 1 (see Table 3 and Additional file 3). We summarize the participant flow through the study in Fig. 5. The outcomes for effectiveness are presented in Table 6.

**Table 6:** Effectiveness outcomes and timing of measurements

Outcome	Outcome Indicators	Measures	Tools	Sources	Timing of measures			
					T0^**a**^	T1^**b**^	T2^**c**^	T3^**d**^
Primary Outcome	Self-management behavior	Survey	Chronic Kidney Disease Self-Management instrument [38, 56]	Patient	X	X	X	X
Secondary outcome	Biomedical status	Clinical records	Blood pressure, Bodyweight, Glomerular filtration rate, Serum albumin, Length, Serum calcium, Serum phosphate, Serum hemoglobin, Sodium and protein in 24 h urine, albumin/creatinine ratio, Cholesterol, High-density lipoprotein, Low-density lipoprotein, Triglycerides, Hemoglobin A1C, Complications	Patient	X	X	X	X
	Self-efficacy	Survey	Chronic Kidney Disease Self-efficacy scale [56, 59]	Patient	X	X	X	X
	Illness perception	Survey	Brief Illness Perception Questionnaire [37, 60]	Patient	X			X
	Quality of life	Survey	The Kidney Disease Quality of Life 36-item short-form survey [61–63]	Patient	X			X
	Mental health	Survey	Hospital Anxiety and Depression Scale [64]	Patient	X	X		X
	Hospital admission	Survey	The time to first acute hospital admission with an exacerbation of CKD or death due to CKD within 9 months after randomization	Patient				X
	Healthcare utilization	Survey	Number of hospitalizations and emergency room visits of patients, primary and secondary care visits	Patient		X		X
	Cost-benefit analysis	Records	All costs delivering the interventions (e.g., materials used in the interventions)	Program, intervention group		X		X
		Records	Medical cost (e.g., cost of treatment, hospitalization rates minored as monetary terms)	Patient		X		X

^a^At baseline

^b^Three months post-randomization

^c^Six months post-randomization

^d^Nine months post-randomization

A cluster-randomized trial will be performed. This means that health-care providers within different units of the Department of Nephrology will be randomized to either the intervention arm or the control arm using a computer random number generator. Also, we will use block randomization of patients. A biostatistician blind to the study conditions will randomly allocate patients to the intervention (group 1) or control group (group 2) by using a computer-based block randomization procedure. The number of patients in each condition with pre-determined characteristics (i.e., CKD stage, age, gender) will be predefined, and block sizes of 4 and 6 will be used to ensure equal allocation to the two groups. Only the biostatistician will know the block sizes. Thereafter, researchers and patients will be notified of the allocation. The care providers delivering the intervention cannot be blind to the intervention, but will not collect data or analyze outcomes. Those conducting statistical analyses will be blind to group allocation until the evaluation is completed.

##### Implementation study

Patients with CKD, as well as care providers in the intervention group, will participate in the process evaluation to evaluate implementation integrity (fidelity) and determinants of implementation. Implementation outcomes on the patient level as well as care provider level will be evaluated, see the further paragraph about details of outcomes of implementation. A research assistant who will not involve in the RCT study will collect data within process evaluation.

#### Sample size calculation

##### Effectiveness; RCT

Based on previous literature [65], we expect the mean CKD self-management score of patients in the intervention group to be approximately 102 ± 20.53 after the 9-month intervention period. When assuming an 80% power and a two-sided significance level of 0.05, the sample size required in each group is 38 patients [66]. Considering a dropout rate of 30% to follow-up, the sample size of patients in this study needs to be 98 patients in total (49 in the intervention and 49 in the comparison group).

##### Implementation study

All patients with CKD in the intervention group will be invited to complete the survey. Also, patients with CKD and care providers in the intervention group will be invited and interviewed either face to face or by telephone for the process evaluation. We will use “purposive and convenience sampling” to ensure diversity (e.g., CKD stage age, gender) of our sample, especially when there are many patients who would like to join the interview study and choices concerning participation need to be made. If only a few patients indicate that they want to participate in the interview study, we will use snowball sampling [41] to recruit more participants. Via snowball sampling, current participants will be asked if they know any other individuals who could participate in the study. As there are no defined rules for calculating sample size in qualitative studies [42], we expect to conduct a minimum of 10–15 interviews minimum with patients with CKD and care providers per group based on previous literature and our experience in previous studies. The definitive sample size for the interviews will be determined based on when data saturation is achieved through the preliminary analysis of the data [43].

#### Outcomes measures & data collection

##### Outcomes for the RCT evaluating the effectiveness

We plan to evaluate:

- patients’ physical outcomes including biomedical measures,
- patients’ lifestyle and psychosocial functioning including self-efficacy, perceptions about CKD, quality of life, anxiety and depression status,
- hospital admission, health care utilization, and cost-benefit

A trained research assistant will conduct data collection, and the intervention monitoring group will supervise the data collection process. We will invite participants in both the intervention and comparison group to visit the Department of Nephrology at the First Affiliated Hospital of Zhengzhou University for data collection at baseline (T0), 3 months (T1), 6 months (T2) and 9 months (T3) post-randomization. At baseline, we will collect demographic data, including age, race, income, education, marital status, work type of participants. To avoid dropping out of participants, if participants cannot come to the hospital, data will then be collected via telephone interview. Table 6 provides details on the proposed outcome measures and timing of the measures. The operationalization of outcomes and descriptions of the measurement tools used are detailed in Additional file 5.

##### Outcomes for implementation integrity (fidelity), and determinants of implementation

The process evaluation will be based on the RE-AIM framework [67]. The RE-AIM model is used to comprehensively measure the public health impact of research conducted in real-world settings [67]. Four dimensions (with the *Effectiveness* domain being applicable above)—Reach *(refers to the proportion of patients with CKD and care providers reached by our program)*, Adoption *(refers to the proportion of participants who use our intervention)*, Implementation *(refer to completion as well as fidelity to the protocol)*, and Maintenance will be used to evaluate the implementation only in the intervention group. We will collect the implementation outcome measurements throughout the 9-month trial. The outcome measures for each dimension of the RE-AIM model are as described in Table 7.

**Table 7:** Implementation outcomes (intervention group only)

Outcome	Outcome Indicators	Measures	Tools	Sources
Reach	Number of patients (eligible, excluded, enrolled)	Records	The proportion of patients eligible to use our intervention program, excluded, invited, and enrolled	Patient
	Number of health care providers (eligible, excluded, enrolled)	Records	The proportion of care professionals eligible to use our intervention program, excluded, invited, and enrolled	Care provider
	Characteristics of participating patients	Records	Comparing participating patients to the target population on key clinical characteristics (e.g., disease stage)	Patient
	Qualitative assessment-reach	Interview	The barriers/facilitators to study participation	Patient
Adoption	Characteristics of participating care providers	Records	Comparing participating care providers to the target population on key characteristics (e.g., work type)	Care provider
	Use of program	Records	Frequency of materials or Medical Dashboard used	Patient, care provider
	Qualitative assessment-adoption	Interview	The appropriateness, comfort, relative advantage, and credibility of the intervention	Patient, care provider
Implementation	Implementation completion	Interview, observation	The implementation completion tasks will be made as a checklist, and the completion of the task and the length of time to finish will be checked.	Patient, care provider
	Acceptability and feasibility of the intervention	Interview	Experiences and perceptions of the intervention	Patients, care provider, research assistant
Maintenance	Follow up on the use of Medical Dashboard	Records	The use of intervention to assess long-term maintenance	Records
	Qualitative assessment-maintenance	Interview	Perceptions of the integration of intervention in health facilities	Patient, care provider

We will use the Measurement Instrument for Determinants of Innovations questionnaire [68, 69] to evaluate the determinants of implementation. Also, individual interviews with stakeholders (e.g. patients, care providers) will be conducted to learn more about the usability and feasibility of Medical Dashboard, its potential for wide-scale implementation, and barriers and facilitators to implementation. We will categorize the determinants identified from this mixed-method study according to Fleuren Framework [70].

### Data analysis

#### Qualitative data analysis

A Framework Method [71] will be used to guide our qualitative analysis. We will structure the qualitative data in a matrix output formed by rows (cases), columns (codes), and ‘cells’ (summarized data). We will follow the Consolidated Criteria for Reporting Qualitative Health Research (COREQ) to ensure quality and validity [72]. The preliminary analysis with proposed codes and a data saturation grid [43] will be performed to determine when data saturation is reached. Also, the codes developed, and results of the preliminary analysis will be taken into account when performing Framework Method analysis.

##### Stage A: transcribing

All audio-taped interviews will be anonymized and transcribed verbatim in Chinese. Long pauses and interruptions (relevant to the study subject) will be noted within the text. Additionally, all participants’ names will be replaced by an ID number. Any names mentioned during the interview will not be transcribed. One researcher will perform transcription, and another will check them to ensure content accuracy.

##### Stage B: familiarization

Two researchers HS (female, 28 years old, a PhD student in the field of public health and primary care) and WW (female, 23 years old, Master of Science in Nursing) will independently read all transcriptions and make contextual/reflective notes to become familiar with the whole data set.

##### Stage C: development of an analytical framework& coding

Atlas.ti for Windows version 7.5.18 (Scientific Software development, Berlin) will be used to analyze our data. Our study includes four qualitative research parts. These are research into the (1) needs, beliefs, perceptions toward CKD and self-management (phase 1); (2) needs, beliefs, perceptions toward eHealth self-management interventions in CKD (phase 1); (3) the acceptability and usability of intervention components (phase 3); (4) determinants of implementation of eHealth self-management interventions (phase 3). Therefore, based on prior literature in which specific theoretical frameworks were used for similar research questions [73–77], we will develop four distinct initial coding trees. For the first and second research questions, we will develop two coding trees based on the adapted version of the theoretical framework of Brakema et al., (submitted) and the TDF [78]. The Technology Acceptance Model [79] will be used to develop the coding tree for evaluating the acceptability and usability of intervention components. Also, the Fleuren framework [70] will be used to develop the coding tree for determinants of implementation of eHealth self-management interventions. The second researcher and third researcher will check the coding tree developed and make amendments if necessary. One researcher will then independently code two or three transcripts using the coding tree, and add new codes if the textual abstracts identified do not fit with the existing set of codes. Then, this researcher will meet with the second researcher and discuss the newly added codes. New codes will be added into the coding tree, and if needed, related codes will be grouped into categories. Thus, the process will be repeated until no new codes arise.

The final coding tree will be checked and approved by the second researcher and the third researcher. This coding tree will include codes and categories; all codes and categories will be operationalized, and relevant examples will be provided.

The finalized coding tree will then be applied to each transcript. One researcher will go through each transcript, highlight the meaningful textual abstracts, and assign the appropriate code from the final coding tree. Then, all codes assigned will be verified by the second researcher. All coding differences will be discussed until consensus is reached.

##### Stage D: charting data into the framework matrix

Data will be charted into matrices per research question identified by two researchers using Microsoft Excel 2010. The matrix will comprise of one row per participant and one column per code. Interesting or illustrative quotations will be added to the matrices.

##### Stage E: interpreting the data

Overarching themes will be generated from codes derived from the data set by reviewing the matrix and making connections within and between participants and codes. Relations, connections, and causality will be further explored and interpreted, and conclusions will be drawn.

As for data derived from observations, all checklists will be digitalized and transported to Microsoft Excel 2010. Also, all written filed notes will be digitalized and will be taken into account to triangulate data collected from other methods.

#### Quantitative data analysis

All quantitative analyses will be performed using SPSS version 23 (IBM, Armonk, NY, USA). We will enter the quantitative data into Microsoft Excel 2010 and calculate descriptive statistics such as the mean, standard deviation, median, and range of linear variables, and frequencies and percentages of categorical variables.

To gain insight into the needs, beliefs, perceptions of patients with CKD towards disease (self-management) and the use of eHealth interventions in phase 1, we will use the descriptive statistics to describe patients’ demographic characteristics, BIPQ scores, CKD-SM score, and C-eHEALS scores. Also, we will conduct secondary analysis using (1) independent *t*-tests for normally distributed continuous variables, (2) Mann–Whitney U-tests for nonnormally distributed variables and (3) Chi-squared or Fisher’s exact tests for categorical variables to compare the difference between certain types of different groups of patients with CKD (e.g., age, gender, disease stage) and BIPQ scores, CKD SM score and C-eHEALS scores. *P*-values < 0.05 and odds ratios with a 95% confidence interval excluding one will be considered statistically significant.

In phase 3, one of the primary hypothesis is that patients in the intervention group, when compared to the comparison group, will demonstrate (statistically) significant improvement in self-management behavior at 9 months post-randomization. Secondary hypotheses are that the intervention group when compared to patients in the comparison group, will demonstrate (statistically) significant improvement in biomedical status, self-efficacy, illness perception, mental health, quality of life, hospital admission, healthcare utilization and cost-benefit analysis at the timing of measurement. All primary statistical analyses will be conducted using intent-to-treat methods. The primary goal of statistical analyses is to examine and compare trends over time in the primary outcome. We will replicate this analytic approach for other secondary outcomes; secondary analyses will examine trends over time for biomedical status, self-efficacy, illness perception, mental health, quality of life, hospital admission, healthcare utilization, and cost-benefit analysis. We will use longitudinal, mixed-model analyses to test the hypotheses. Exploratory analyses will assess the impact of the intervention on primary and secondary outcomes for patients.

#### Mixed analysis of literature review, qualitative and quantitative data by triangulation

We will conduct a combined analysis by merging results of all data analysis; from the review, quantitative and qualitative research [80]. In phase 1, the quantitative results and review results will triangulate the qualitative results to gain insight into the perception of disease, self-management behavior, eHealth literacy, and needs towards CKD self-management. To this end, we will develop a thematic matrix [81] that includes participants’ characteristics and data derived from surveys and emerging themes from our qualitative results to summarize patients’ illness perception, self-management behavior, and eHealth literacy. Also, another thematic matrix will be developed that includes study characteristics of scoping review and data derived from review results and emerging themes from our qualitative results to summarize the needs of patients and care providers towards CKD self-management. These results will be combined to inform the development of ‘Medical Dashboard’ based intervention (plan) in phase 2. For instance, if the review and qualitative results show that health education is needed to improve CKD self-management behaviors, we will develop the educational intervention components in the future intervention plan. In phase 3, we will use the results collected from the qualitative interviews to help interpret the quantitative results from the trial. Qualitative results will, therefore, be used to expand upon the results of this trial to understand the implementation process as experienced by participants. For instance, the questionnaire of determinants of implementation will be matched with the qualitative research on determinants of implementation.

## Discussion

Some research has shown that eHealth based self-management interventions in CKD can help to improve health-related outcomes. However, evidence on the effectiveness of CKD eHealth based self-management interventions is still inconclusive [21]. Thus, our study will gain insights into the development of theoretically based, and target population tailored implementation of eHealth based self-management interventions to improve CKD care. Our study will add knowledge on the implementation research of eHealth self-management interventions in CKD care, with fitting with the needs and priorities expressed by patients and health care professionals. Also, this study will add evidence of the effectiveness of eHealth based self-management interventions on CKD health outcomes.

There are some strengths to our research. First, we will use an innovative hybrid design to concurrently study the effectiveness and implementation of the tailored ‘Medical Dashboard’ self-management intervention in CKD care. The hybrid designs can test the implementation process by looking inside the so-called “black box” to see what happens in the intervention implementation and how that could affect intervention outcomes [82, 83]. Therefore, hybrid designs can provide the potential to speed the translation of intervention findings into routine practice by optimizing the implementation process [54]. In addition, the triangulation of both quantitative and qualitative results allows researchers to understand the implementation process and intervention effectiveness from multiple perspectives, different types of causal pathways, and multiple types of outcome, thereby strengthening the validity of intervention effects [80, 82]. Second, the robust theory will be used to guide the process of intervention development. The IM method ensures a theory-based approach from the recognition of a need or problem to the identification of a solution and intervention testing. To translate interventions into different contexts (e.g., health care system, population), it is essential to optimize the intervention fit with the needs and priorities expressed by the target population. IM was successfully applied in the development of self-management interventions for osteoarthritis and chronic low back pain [75], and children with CKD [84, 85]. Also, the RE-AIM framework as utilized in this study provides systematic guidance on how to evaluate the intervention effect on the process and outcome level. A major limitation of this study is that we only perform the study within one hospital in China. Hence, findings may not be immediately generalizable to other health system contexts in China where the access to eHealth technology is (more) limited. Also, the transferability of developed Medical Dashboard self-management intervention to routine clinical practice in primary care may be limited and needs further exploration. Additionally, barriers to the adoption of Medical Dashboard may be technical issues (e.g., connectivity issues) or a low level of eHealth literacy of participants. To address these challenges, we will include intervention components such as the provision of ongoing technical support and eHealth literacy training in the intervention plan.

In conclusion, our study will result in the delivery of a culturally tailored, standardized eHealth self-management intervention for patients with CKD in China, which has the potential to optimize patients’ self-management skills and improve health status and quality of life. Also, this study can serve as proof of concept for the use of IM and a hybrid type 2 trial design to evaluate the implementation and effectiveness of eHealth self-management interventions. Moreover, it will inform future research on the tailoring and translation of evidence-based eHealth self-management interventions in various contexts.

## Supplementary Information

**Additional file 1.** Core intervention components of Medical Dashboard and evidence base.

**Additional file 2.** Search strategy.

**Additional file 3.** Detailed methods and relevant materials of the mixed-method study.

**Additional file 4.** SPIRIT 2013 Statement: Defining standard protocol items for clinical trials.

**Additional file 5.** The operationalization of outcomes and descriptions of the measurement tools used in RCT.

## Data Availability

Data sharing is not applicable to this article as no datasets were generated or analysed during the current study. Once available, study data are available from the corresponding author on reasonable request.

## Abbreviations

CKD: Chronic kidney disease
GFR: Glomerular filtration rate
eHealth: Electronic health
BP: Blood pressure
LUMC: Leiden University Medical Center
RCT: Randomized controlled trial
IM: Intervention Mapping
BIPQ: Brief Illness Perception Questionnaire
CKD-SM: Chronic Kidney Disease Self-management instrument
C-eHEALS: Chinese eHealth Literacy Scale
KDIGO: Kidney Disease Improving Global Outcomes
TDF: Theoretical Domains Framework
CKD-SE: Chronic Kidney Disease Self-efficacy
KDQOL-36: The Kidney Disease Quality of Life 36-item short-form survey
HADS: Hospital Anxiety and Depression Scale
COREQ: Consolidated Criteria for Reporting Qualitative Health Research
