# Supplementary material for: Development and evaluation of an eHealth self-management intervention for patients with chronic kidney disease in China: protocol for a mixed-method hybrid type 2 trial
Source: BMC Nephrol. 2020 Nov 19;21:495. doi: 10.1186/s12882-020-02160-6 (PMC7678219; doi:10.1186/s12882-020-02160-6)
Supplement: Supplementary file 1 — Additional file 1. Core intervention components of Medical Dashboard and evidence base. [file 12882_2020_2160_MOESM1_ESM.docx]

**Additional file 1. Core intervention components of Medical Dashboard and evidence base**

The intervention consists of the following core intervention components (i.e. active intervention parts that support self-management behaviour, including elements defined as what is provided to the user, how these elements are delivered, and the subsequent intervention workflow defined as when they are delivered), see Table S1. Supporting evidence base of Medical Dashboard intervention are presented below the Table S1 [1-6].

**Table S1 Core intervention components of Medical Dashboard self-management intervention**

| **Intervention Component** | **Details** |
| --- | --- |
| Personal coaches | Patients are coupled with one of four personal coaches: three health psychologists and one dietician. |
| Motivational interviewing | Patients are provided with a one-hour individual motivational interview at the patient’s hospital, which focuses on discussing barriers, benefits, and strategies for self-management; setting personal goals, and strengthening intrinsic motivation and self-efficacy. |
| Education | Patients are provided with education, a kidney-friendly cookbook, instructions for self-monitoring BP (using a Microlife Watch BP Home device), dietary intake (using an online food diary), and 24-hour urinary sodium excretion (using an innovative point-of-care chip-device). |
| Self-monitoring | Depending on patients’ preferences, patients are instructed to take measurements, and enter the results of home measurements via the “self-care” website [www.bonstat.nl](http://www.bonstat.nl), for instance, blood pressure, weight and glucose. The measurements from this website can link to the Medical Dashboard. |
| Combination of home and hospital measurements | The measurements that patients take at home and the crucial measurements from the hospital are grouped in the Medical Dashboard. The measurement results with patients’ personal and general goals can be compared. |
| Coaching | Following these self-monitoring measurements, patients are provided with feedback by telephone from their coach. The discussion will focus on the progression, achievements, barriers, and possible solutions of self-management. |
| Information support | Patients are provided, if they want, with information support regarding social support, refusal skills, medication adherence strategies, physical exercise,  healthy eating, smoking, and alcohol intake. |

**Reference**

1. Wang W, van Lint CL, Brinkman WP, Rövekamp TJM, van Dijk S, van der Boog PJM, et al. Renal transplant patient acceptance of a self management support system. [BMC Med Inform Decis Mak.](https://www.ncbi.nlm.nih.gov/pubmed/?term=Renal+transplant+patient+acceptance+of+a+self-management+support+system) 2017;17:58.
2. [van Lint CL](https://www.ncbi.nlm.nih.gov/pubmed/?term=van%20Lint%20CL%5BAuthor%5D&cauthor=true&cauthor_uid=26673985), [van der Boog PJ](https://www.ncbi.nlm.nih.gov/pubmed/?term=van%20der%20Boog%20PJ%5BAuthor%5D&cauthor=true&cauthor_uid=26673985), [Wang W](https://www.ncbi.nlm.nih.gov/pubmed/?term=Wang%20W%5BAuthor%5D&cauthor=true&cauthor_uid=26673985),  [Brinkman WP](https://www.ncbi.nlm.nih.gov/pubmed/?term=Brinkman%20WP%5BAuthor%5D&cauthor=true&cauthor_uid=26673985), [Rövekamp TJ](https://www.ncbi.nlm.nih.gov/pubmed/?term=R%C3%B6vekamp%20TJ%5BAuthor%5D&cauthor=true&cauthor_uid=26673985), [Neerincx MA](https://www.ncbi.nlm.nih.gov/pubmed/?term=Neerincx%20MA%5BAuthor%5D&cauthor=true&cauthor_uid=26673985), et al. Patient experiences with self-monitoring renal function after renal transplantation: results from a single-center prospective pilot study. [Patient Prefer Adherence.](https://www.ncbi.nlm.nih.gov/pubmed/?term=Patient+experiences+with+self-monitoring+renal+function+after+renal+transplantation%3A+results+from+a+single-center+prospective+pilot+study) 2015;9:1721-31.
3. [van Lint C](https://www.ncbi.nlm.nih.gov/pubmed/?term=van%20Lint%20C%5BAuthor%5D&cauthor=true&cauthor_uid=28951385), [Wang W](https://www.ncbi.nlm.nih.gov/pubmed/?term=Wang%20W%5BAuthor%5D&cauthor=true&cauthor_uid=28951385), [van Dijk S](https://www.ncbi.nlm.nih.gov/pubmed/?term=van%20Dijk%20S%5BAuthor%5D&cauthor=true&cauthor_uid=28951385),  [Brinkman WP](https://www.ncbi.nlm.nih.gov/pubmed/?term=Brinkman%20WP%5BAuthor%5D&cauthor=true&cauthor_uid=28951385), [Rövekamp TJ](https://www.ncbi.nlm.nih.gov/pubmed/?term=R%C3%B6vekamp%20TJ%5BAuthor%5D&cauthor=true&cauthor_uid=28951385), [Neerincx MA](https://www.ncbi.nlm.nih.gov/pubmed/?term=Neerincx%20MA%5BAuthor%5D&cauthor=true&cauthor_uid=28951385), et al. Self Monitoring Kidney Function Post Transplantation: Reliability of Patient-Reported Data. [J Med Internet Res.](https://www.ncbi.nlm.nih.gov/pubmed/?term=Self-Monitoring+Kidney+Function+Post+Transplantation%3A+Reliability+of+Patient-Reported+Data) 2017; doi: 10.2196/jmir.7542.
4. [Meuleman Y](https://www.ncbi.nlm.nih.gov/pubmed/?term=Meuleman%20Y%5BAuthor%5D&cauthor=true&cauthor_uid=27993433), [Hoekstra T](https://www.ncbi.nlm.nih.gov/pubmed/?term=Hoekstra%20T%5BAuthor%5D&cauthor=true&cauthor_uid=27993433), [Dekker FW](https://www.ncbi.nlm.nih.gov/pubmed/?term=Dekker%20FW%5BAuthor%5D&cauthor=true&cauthor_uid=27993433), [Navis G](https://www.ncbi.nlm.nih.gov/pubmed/?term=Navis%20G%5BAuthor%5D&cauthor=true&cauthor_uid=27993433), [Vogt L](https://www.ncbi.nlm.nih.gov/pubmed/?term=Vogt%20L%5BAuthor%5D&cauthor=true&cauthor_uid=27993433), [van der Boog PJM](https://www.ncbi.nlm.nih.gov/pubmed/?term=van%20der%20Boog%20PJM%5BAuthor%5D&cauthor=true&cauthor_uid=27993433), et al. Sodium Restriction in Patients With CKD: A Randomized Controlled Trial of Self-management Support. [Am J Kidney Dis. 2017;69: 576-586](https://www.ncbi.nlm.nih.gov/pubmed/?term=Sodium+Restriction+in+Patients+With+CKD%3A+A+Randomized+Controlled+Trial+of+Self-management+Support).
5. van Lint CL, van der Boog PJ, Romijn FP, Schenk PW, van Dijk S, Rövekamp TJ, et al. Application of a point of care creatinine device for trend monitoring in kidney transplant patients: fit for purpose. [Clin Chem Lab Med. 2015;53:](https://www.ncbi.nlm.nih.gov/pubmed/?term=Application+of+a+point+of+care+creatinine+device+for+trend+monitoring+in+kidney+transplant+patients%3A+fit+for+purpose%3F) 1547-56.
6. [Humalda JK](https://www.ncbi.nlm.nih.gov/pubmed/?term=Humalda%20JK%5BAuthor%5D&cauthor=true&cauthor_uid=31955921), [Klaassen G](https://www.ncbi.nlm.nih.gov/pubmed/?term=Klaassen%20G%5BAuthor%5D&cauthor=true&cauthor_uid=31955921), [de Vries H](https://www.ncbi.nlm.nih.gov/pubmed/?term=de%20Vries%20H%5BAuthor%5D&cauthor=true&cauthor_uid=31955921), [Meuleman Y](https://www.ncbi.nlm.nih.gov/pubmed/?term=Meuleman%20Y%5BAuthor%5D&cauthor=true&cauthor_uid=31955921), [Verschuur LC](https://www.ncbi.nlm.nih.gov/pubmed/?term=Verschuur%20LC%5BAuthor%5D&cauthor=true&cauthor_uid=31955921), [Straathof EJM](https://www.ncbi.nlm.nih.gov/pubmed/?term=Straathof%20EJM%5BAuthor%5D&cauthor=true&cauthor_uid=31955921), et al. A Self-management Approach for Dietary Sodium Restriction in Patients With CKD: A Randomized Controlled Trial. Am J Kidney Dis. 2020;  doi: 10.1053/j.ajkd.2019.10.012.
