# Supplementary material for: Development and evaluation of an eHealth self-management intervention for patients with chronic kidney disease in China: protocol for a mixed-method hybrid type 2 trial
Source: BMC Nephrol. 2020 Nov 19;21:495. doi: 10.1186/s12882-020-02160-6 (PMC7678219; doi:10.1186/s12882-020-02160-6)
Supplement: Supplementary file 2 — Additional file 2. Search strategy. [file 12882_2020_2160_MOESM2_ESM.docx]

**Additional file 2. Search strategy**

**(**(("support needs"[tw] OR "support need"[tw] OR "care needs"[tw] OR "care need"[tw] OR "mental health needs"[tw] OR "mental health need"[tw] OR "advance care planning needs"[tw] OR "care planning needs"[tw] OR "information need"[tw] OR "information needs"[tw] OR "unmet health care need"[tw] OR "unmet health care needs"[tw] OR "unmet healthcare need"[tw] OR "unmet healthcare needs"[tw] OR "unmet need"[tw] OR "unmet needs"[tw] OR "health care need"[tw] OR "health care needs"[tw] OR "healthcare need"[tw] OR "healthcare needs"[tw] OR "Needs Assessment"[Mesh] OR "Needs Assessment"[tw] OR "need assessment"[tw] OR "perceived need"[tw] OR "perceived needs"[tw] OR "unperceived need"[tw] OR "unperceived needs"[tw] OR "assessing need"[tw] OR "assessing needs"[tw] OR "assessed need"[tw] OR "assessed needs"[tw] OR "supportive care need"[tw] OR "supportive care needs"[tw] OR "Hope"[Mesh] OR "hope"[tw] OR "hopes"[tw] OR "hopefulness"[tw] OR "patient experience"[tw] OR "patients experience"[tw] OR "patient's experience"[tw] OR "patients' experience"[tw] OR "patient experiences"[tw] OR "patients experiences"[tw] OR "patient's experiences"[tw] OR "patients' experiences"[tw] OR "personal experience"[tw] OR "personal experiences"[tw] OR "Patient Satisfaction"[Mesh] OR "patient satisfaction"[tw] OR "patients satisfaction"[tw] OR "patient's satisfaction"[tw] OR "patients' satisfaction"[tw] OR "patient satisfactions"[tw] OR "patients satisfactions"[tw] OR "patient's satisfactions"[tw] OR "patients' satisfactions"[tw] OR "personal satisfaction"[tw] OR "personal satisfactions"[tw] OR "patient perception"[tw] OR "patients perception"[tw] OR "patient's perception"[tw] OR "patients' perception"[tw] OR "patient perceptions"[tw] OR "patients perceptions"[tw] OR "patient's perceptions"[tw] OR "patients' perceptions"[tw] OR "personal perception"[tw] OR "personal perceptions"[tw] OR "Patient Preference"[Mesh] OR "patient preference"[tw] OR "patients preference"[tw] OR "patient's preference"[tw] OR "patients' preference"[tw] OR "patient preferences"[tw] OR "patients preferences"[tw] OR "patient's preferences"[tw] OR "patients' preferences"[tw] OR "personal preference"[tw] OR "personal preferences"[tw] OR "patient utility"[tw] OR "patients utility"[tw] OR "patient's utility"[tw] OR "patients' utility"[tw] OR "patient utilities"[tw] OR "patients utilities"[tw] OR "patient's utilities"[tw] OR "patients' utilities"[tw] OR "personal utility"[tw] OR "personal utilities"[tw] OR "patient attitude"[tw] OR "patients attitude"[tw] OR "patient's attitude"[tw] OR "patients' attitude"[tw] OR "patient attitudes"[tw] OR "patients attitudes"[tw] OR "patient's attitudes"[tw] OR "patients' attitudes"[tw] OR "personal attitude"[tw] OR "personal attitudes"[tw] OR "patient expectation"[tw] OR "patients expectation"[tw] OR "patient's expectation"[tw] OR "patients' expectation"[tw] OR "patient expectations"[tw] OR "patients expectations"[tw] OR "patient's expectations"[tw] OR "patients' expectations"[tw] OR "personal expectation"[tw] OR "personal expectations"[tw] OR "patient willing"[tw] OR "patients willing"[tw] OR "patient's willing"[tw] OR "patients' willing"[tw] OR "patient willings"[tw] OR "patients willings"[tw] OR "patient's willings"[tw] OR "patients' willings"[tw] OR "personal willing"[tw] OR "personal willings"[tw] OR "patient willingness"[tw] OR "patients willingness"[tw] OR "patient's willingness"[tw] OR "patients' willingness"[tw] OR "patient willingnesss"[tw] OR "patients willingnesss"[tw] OR "patient's willingnesss"[tw] OR "patients' willingnesss"[tw] OR "personal willingness"[tw] OR "personal willingnesss"[tw] OR "patient value"[tw] OR "patients value"[tw] OR "patient's value"[tw] OR "patients' value"[tw] OR "patient values"[tw] OR "patients values"[tw] OR "patient's values"[tw] OR "patients' values"[tw] OR "personal value"[tw] OR "personal values"[tw] OR "patient perspective"[tw] OR "patients perspective"[tw] OR "patient's perspective"[tw] OR "patients' perspective"[tw] OR "patient perspectives"[tw] OR "patients perspectives"[tw] OR "patient's perspectives"[tw] OR "patients' perspectives"[tw] OR "personal perspective"[tw] OR "personal perspectives"[tw] OR "patient view"[tw] OR "patients view"[tw] OR "patient's view"[tw] OR "patients' view"[tw] OR "patient views"[tw] OR "patients views"[tw] OR "patient's views"[tw] OR "patients' views"[tw] OR "personal view"[tw] OR "personal views"[tw] OR "patient understanding"[tw] OR "patients understanding"[tw] OR "patient's understanding"[tw] OR "patients' understanding"[tw] OR "patient understandings"[tw] OR "patients understandings"[tw] OR "patient's understandings"[tw] OR "patients' understandings"[tw] OR "personal understanding"[tw] OR "personal understandings"[tw] OR "patient knowledge"[tw] OR "patients knowledge"[tw] OR "patient's knowledge"[tw] OR "patients' knowledge"[tw] OR "patient knowledges"[tw] OR "patients knowledges"[tw] OR "patient's knowledges"[tw] OR "patients' knowledges"[tw] OR "personal knowledge"[tw] OR "personal knowledges"[tw] OR "Health Literacy"[Mesh] OR "Health Literacy"[tw]) AND **("Renal Insufficiency, Chronic"[majr] OR "chronic kidney disease"[ti] OR "chronic kidney diseases"[ti] OR "chronic renal disease"[ti] OR "chronic renal diseases"[ti] OR "chronic kidney failure"[ti] OR "chronic renal failure"[ti] OR "frasier syndrome"[ti] OR "chronic kidney"[ti] OR "chronic renal"[ti] OR "CKD"[ti] OR (("Kidney Diseases"[majr] OR "kidney disease"[ti] OR "kidney diseases"[ti] OR "renal disease"[ti] OR "renal diseases"[ti]) AND ("Chronic Disease"[majr] OR "chronic"[ti] OR chronic*[ti])) OR "Kidney Diseases"[majr] OR "kidney disease"[ti] OR "kidney injury"[ti] OR "kidney disorder"[ti] OR "kidney insufficiency"[ti] OR "kidney failure"[ti] OR "kidney transplantation"[ti] OR "kidney transplant"[ti] OR "renal disease"[ti] OR "renal injury"[ti] OR "renal disorder"[ti] OR "renal insufficiency"[ti] OR "renal failure"[ti] OR "renal transplantation"[ti] OR "renal transplant"[ti] OR "dialysis"[ti] OR "kidney diseases"[ti] OR "kidney injuries"[ti] OR "kidney disorders"[ti] OR "kidney transplants"[ti] OR "renal diseases"[ti] OR "renal injuries"[ti] OR "renal disorders"[ti] OR "renal transplants"[ti] OR "dialysis"[ti] OR "Renal Dialysis"[majr] OR "hemodialysis"[ti] OR "hemodialysis"[ti])) OR** (("support needs"[ti] OR "support need"[ti] OR "care needs"[ti] OR "care need"[ti] OR "mental health needs"[ti] OR "mental health need"[ti] OR "advance care planning needs"[ti] OR "care planning needs"[ti] OR "information need"[ti] OR "information needs"[ti] OR "unmet health care need"[ti] OR "unmet health care needs"[ti] OR "unmet healthcare need"[ti] OR "unmet healthcare needs"[ti] OR "unmet need"[ti] OR "unmet needs"[ti] OR "health care need"[ti] OR "health care needs"[ti] OR "healthcare need"[ti] OR "healthcare needs"[ti] OR "Needs Assessment"[majr] OR "Needs Assessment"[ti] OR "need assessment"[ti] OR "perceived need"[ti] OR "perceived needs"[ti] OR "unperceived need"[ti] OR "unperceived needs"[ti] OR "assessing need"[ti] OR "assessing needs"[ti] OR "assessed need"[ti] OR "assessed needs"[ti] OR "supportive care need"[ti] OR "supportive care needs"[ti] OR "Hope"[majr] OR "hope"[ti] OR "hopes"[ti] OR "hopefulness"[ti] OR "patient experience"[ti] OR "patients experience"[ti] OR "patient's experience"[ti] OR "patients' experience"[ti] OR "patient experiences"[ti] OR "patients experiences"[ti] OR "patient's experiences"[ti] OR "patients' experiences"[ti] OR "personal experience"[ti] OR "personal experiences"[ti] OR "Patient Satisfaction"[majr] OR "patient satisfaction"[ti] OR "patients satisfaction"[ti] OR "patient's satisfaction"[ti] OR "patients' satisfaction"[ti] OR "patient satisfactions"[ti] OR "patients satisfactions"[ti] OR "patient's satisfactions"[ti] OR "patients' satisfactions"[ti] OR "personal satisfaction"[ti] OR "personal satisfactions"[ti] OR "patient perception"[ti] OR "patients perception"[ti] OR "patient's perception"[ti] OR "patients' perception"[ti] OR "patient perceptions"[ti] OR "patients perceptions"[ti] OR "patient's perceptions"[ti] OR "patients' perceptions"[ti] OR "personal perception"[ti] OR "personal perceptions"[ti] OR "Patient Preference"[majr] OR "patient preference"[ti] OR "patients preference"[ti] OR "patient's preference"[ti] OR "patients' preference"[ti] OR "patient preferences"[ti] OR "patients preferences"[ti] OR "patient's preferences"[ti] OR "patients' preferences"[ti] OR "personal preference"[ti] OR "personal preferences"[ti] OR "patient utility"[ti] OR "patients utility"[ti] OR "patient's utility"[ti] OR "patients' utility"[ti] OR "patient utilities"[ti] OR "patients utilities"[ti] OR "patient's utilities"[ti] OR "patients' utilities"[ti] OR "personal utility"[ti] OR "personal utilities"[ti] OR "patient attitude"[ti] OR "patients attitude"[ti] OR "patient's attitude"[ti] OR "patients' attitude"[ti] OR "patient attitudes"[ti] OR "patients attitudes"[ti] OR "patient's attitudes"[ti] OR "patients' attitudes"[ti] OR "personal attitude"[ti] OR "personal attitudes"[ti] OR "patient expectation"[ti] OR "patients expectation"[ti] OR "patient's expectation"[ti] OR "patients' expectation"[ti] OR "patient expectations"[ti] OR "patients expectations"[ti] OR "patient's expectations"[ti] OR "patients' expectations"[ti] OR "personal expectation"[ti] OR "personal expectations"[ti] OR "patient willing"[ti] OR "patients willing"[ti] OR "patient's willing"[ti] OR "patients' willing"[ti] OR "patient willings"[ti] OR "patients willings"[ti] OR "patient's willings"[ti] OR "patients' willings"[ti] OR "personal willing"[ti] OR "personal willings"[ti] OR "patient willingness"[ti] OR "patients willingness"[ti] OR "patient's willingness"[ti] OR "patients' willingness"[ti] OR "patient willingnesss"[ti] OR "patients willingnesss"[ti] OR "patient's willingnesss"[ti] OR "patients' willingnesss"[ti] OR "personal willingness"[ti] OR "personal willingnesss"[ti] OR "patient value"[ti] OR "patients value"[ti] OR "patient's value"[ti] OR "patients' value"[ti] OR "patient values"[ti] OR "patients values"[ti] OR "patient's values"[ti] OR "patients' values"[ti] OR "personal value"[ti] OR "personal values"[ti] OR "patient perspective"[ti] OR "patients perspective"[ti] OR "patient's perspective"[ti] OR "patients' perspective"[ti] OR "patient perspectives"[ti] OR "patients perspectives"[ti] OR "patient's perspectives"[ti] OR "patients' perspectives"[ti] OR "personal perspective"[ti] OR "personal perspectives"[ti] OR "patient view"[ti] OR "patients view"[ti] OR "patient's view"[ti] OR "patients' view"[ti] OR "patient views"[ti] OR "patients views"[ti] OR "patient's views"[ti] OR "patients' views"[ti] OR "personal view"[ti] OR "personal views"[ti] OR "patient understanding"[ti] OR "patients understanding"[ti] OR "patient's understanding"[ti] OR "patients' understanding"[ti] OR "patient understandings"[ti] OR "patients understandings"[ti] OR "patient's understandings"[ti] OR "patients' understandings"[ti] OR "personal understanding"[ti] OR "personal understandings"[ti] OR "patient knowledge"[ti] OR "patients knowledge"[ti] OR "patient's knowledge"[ti] OR "patients' knowledge"[ti] OR "patient knowledges"[ti] OR "patients knowledges"[ti] OR "patient's knowledges"[ti] OR "patients' knowledges"[ti] OR "personal knowledge"[ti] OR "personal knowledges"[ti] OR "Health Literacy"[majr] OR "Health Literacy"[ti]) AND **("Renal Insufficiency, Chronic"[Mesh] OR "chronic kidney disease"[tw] OR "chronic kidney diseases"[tw] OR "chronic renal disease"[tw] OR "chronic renal diseases"[tw] OR "chronic kidney failure"[tw] OR "chronic renal failure"[tw] OR "frasier syndrome"[tw] OR "chronic kidney"[tw] OR "chronic renal"[tw] OR "CKD"[tw] OR (("Kidney Diseases"[mesh] OR "kidney disease"[tw] OR "kidney diseases"[tw] OR "renal disease"[tw] OR "renal diseases"[tw]) AND ("Chronic Disease"[mesh] OR "chronic"[tw] OR chronic*[tw])) OR "Kidney Diseases"[mesh] OR "kidney disease"[tw] OR "kidney injury"[tw] OR "kidney disorder"[tw] OR "kidney insufficiency"[tw] OR "kidney failure"[tw] OR "kidney transplantation"[tw] OR "kidney transplant"[tw] OR "renal disease"[tw] OR "renal injury"[tw] OR "renal disorder"[tw] OR "renal insufficiency"[tw] OR "renal failure"[tw] OR "renal transplantation"[tw] OR "renal transplant"[tw] OR "dialysis"[tw] OR "kidney diseases"[tw] OR "kidney injuries"[tw] OR "kidney disorders"[tw] OR "kidney transplants"[tw] OR "renal diseases"[tw] OR "renal injuries"[tw] OR "renal disorders"[tw] OR "renal transplants"[tw] OR "dialysis"[tw] OR "Renal Dialysis"[Mesh] OR "hemodialysis"[tw] OR "hemodialysis"[tw]))) NOT ("Animals"[mesh] NOT "Humans"[mesh]) AND (english[la] OR dutch[la] OR chinese[la])**
