# Supplementary material for: Development and evaluation of an eHealth self-management intervention for patients with chronic kidney disease in China: protocol for a mixed-method hybrid type 2 trial
Source: BMC Nephrol. 2020 Nov 19;21:495. doi: 10.1186/s12882-020-02160-6 (PMC7678219; doi:10.1186/s12882-020-02160-6)
Supplement: Supplementary file 3 — Additional file 3. Detailed methods and relevant materials of the mixed-method study. [file 12882_2020_2160_MOESM3_ESM.docx]

**Additional file 3. Detailed methods and relevant materials of the mixed-method study**

**Recruitment strategy**

For recruiting health care providers, an online invitation for research participation and details about the study will be sent to the WeChat group of care providers in the Department of Nephrology or explained verbally face to face. For recruiting patients, we will employ two methods. One is that care providers in the Department of Nephrology will deliver flyers with a general introduction of project and contact details of the task lead to patients. The other is that the researchers will put the flyers on the material place in the Department of Nephrology. If one participant expresses interest in our study, he or she will be approached by one of the researchers. The researcher will answer the questions the patients have about the research and ask patients which field study they want to participate (e.g., interview, or survey) If this participant is willing to take part in the study, we will provide him or her with written informed consent matched with the field method. The written informed consent will include a detailed description of the study procedures, risks, and benefits. We will provide verbal explanations that participation will be voluntary and that data will be analysed anonymously and treated confidentially. Participants are encouraged to ask any questions they may have about the study before signing the consent form. Then, informed consent will be obtained from all participants.

**Description of each field method**

The field methods, including face to face interviews, focus group discussions, observations, and survey research, will be employed in this study. We will conduct interviews and surveys with care providers and patients in the care provider’s office as convenient. Also, we will conduct observations in the outpatient clinic or during routine clinical care. The settings for interviews and surveys will ensure the privacy and comfort of the participants.

A) Semi-structured face to face interviews

In face to face semi-structured interview, a trained researcher will conduct this interview according to a semi-structured interview schedule, consisting of a set of standardised interview questions (**Appendix 1a, 1b)**, while allowing flexibility in how and in which order questions are asked. We will conduct interviews in the local language, so using Henan accent or Mandarin. The duration of the interviews will be approximately 45-60 minutes.

B) Focus group discussions

In the focus group discussion, after an introduction about the goal and procedure of this study, the interviewer will ask key questions (**Appendix 2)** which can provide a lead for the discussion. The flow of key questions can be tailored or adapted to the participants’ needs and input during the discussion. In addition, if the discussion shows that more in-depth exploration with a participant about a specific topic would be desirable, we will schedule a subsequent in-depth interview. Each focus group discussion will last approximately one hour.

C) Observations

Observations will be valuable for collecting in-depth detailed data, which might be hard to detect because of possible self-reported bias. Also, it can help triangulate data from other methods and identify potential differences between the observed behaviour of participants during consultations, and the behaviour verbally stated by participants. An observation checklist is structured (**Appendix 3**). We will observe care providers and patients during patient outpatient clinic follow-up or routine care. Before the observation, patients will be informed about the study and asked for informed consent to the presence of an observer in the room when he or she has a consultation with care providers. After the informed consent is obtained, the observation will start.

D) Survey

Three validated measures will be used in the survey research: 1) ‘The Brief Illness Perception Questionnaire’, 2) ‘Chronic kidney disease self-management instrument’, and 3) ‘Chinese eHealth Literacy Scale’. These questionnaires will help triangulate the qualitative data.

**Appendix 1a TOPIC LIST INTERVIEW: Healthcare provider**

Duration Interview: 45-60 min

**A.** Introduction, explanation, consent

**B.** Demographic data (Baseline data):

- Name, sex, age, profession, education (the type of work, years of work experience, type of healthcare facility)
- Personal and cultural background (family situation – and size, composition, religion)

**C.**  Topic lists of belief, perception of disease

1. How do you feel about CKD?

(**Probe**: What is the impact of CKD? and how?)

1. How do you tell patients that they have CKD?

(**Probe**: What will you tell patients that they have CKD? What worries and concerns of their future?)

**D.** Topic lists of belief, perception of self-management

Attitude toward self-management interventions in CKD and behavioural beliefs

1. Do you know self-management?
2. How do you feel of self-management in patients with CKD? And why?

(**Probe**: Do you have any experience with it? How do you think of the advantages of self-management? How do you feel about disadvantages of self-management)

Subjective norms in self-management interventions in CKD and normative beliefs

1. How do you think others’ feelings of self-management?

(**Probe**: government, nurses, patients, patients’ caregivers? Other stakeholders for approving or disapproving?)

Perceived control in self-management interventions in CKD, barriers and facilitators

1. How do you think the possibility of self-management in patients with CKD?

(**Probe**: Do you have confidence in self-management in CKD? Facilitators? barriers)

1. How do you perceive your role in patients’ self-management? What is your responsibility?

(**Probe**: Are you willing to be more involved in the patients’ self-management? Can you explain in which way you want to be involved?)

**E.**  Topic lists of needs toward CKD self-management

Current care

1. Can you tell me about your ongoing care for the patients with CKD?

(**Probe**: What are your roles in clinical care? What types of care will you give to patients? If you follow some kinds of protocols for their care? Are there some protocol contains something related to self-management?)

Challenges and barriers

1. Do you have any barriers in the health care of CKD management?

(**Probe**: Do you have some challenges in patient care? Do you have some barriers to promoting patients’ self-management?)

Needs of CKD self-management

1. Is there anything else that should be done to support you in the clinical care of CKD?

(**Probe**: Do you have some suggestions for the management? What sorts of programs or support should be done to support self-management for patients?)

**F****.**  Topic lists of beliefs, perceptions and needs toward eHealth and Medical Dashboard intervention

eHealth use

1. Do you know about eHealth? Have you ever used eHealth services in health care?
2. **If yes**, what was your experience for eHealth on CKD? What do you think of it?

(**Probe**: motivation? How? Who pays for it? Who offered? When? Benefits? Main risks? advantages/disadvantages?)

**If not**, have you known any services for eHealth? What do you think of it?

(**Probe**: how? Who offered? Who pays for it? When? Benefits? Main risks? advantages/disadvantages?)

Needs toward eHealth

1. What do you want of the eHealth in your health care work and for patients with CKD?

(**probe**: Do you think that eHealth has an important value? Which kind of eHealth do you like or not, why?)

Needs toward Medical Dashboard intervention

1. What is the first thing that comes to mind when you view the material?
2. How do you feel about Medical Dashboard?

(**Probe**: Do you think that it has important value? What are the advantages or disadvantages?)

1. What do you think if it is used in Chinese clinical care?

(**Probe**: What content should be changed and how, why? What do you expect from this program? What are the facilitators or barriers? Who should pay for it?)

1. What thoughts will you have if inviting you about participating in an eHealth program?

(**Probe**: What kind of reasons that influence your intention? What are your expectations, roles of you?)

1. Are there any final points you would like to add on any aspect of the subject, or anything you think we have not covered?

**Appendix 1b TOPIC LIST INTERVIEW: Patients with chronic kidney disease**

Duration Interview: 45-60 min

**A.** Introduction, explanation, consent

**B.** Demographic data:

- Identification number, sex, age, education, profession (e.g., type of work, daily routine)
- Personal and cultural background (e.g., family situation – and size, composition, religion)
- External health-related conditions (e.g., distance to a health care facility), disease stage, comorbidities

**C.** Topic lists of belief, perception of disease

1. How do you feel when you were told that you had CKD?

(**Probe:** What do you think has caused this problem? Did you have any worries or concerns, the most fear?)

1. How do you feel of having CKD?

(**Probe:** What do you know about CKD? What are the changes in your life/impact? How?

How do you cope with these impacts)

**D**. Topic lists of belief, perception of self-management

Attitude toward self-management

1. Do you know self-management?
2. How do you feel about self-management? And why?

(**Probe**: Do you have experience with it? What can you do? How do you think of the advantages of self-management? How do you feel about disadvantages of self-management?)

Subjective norms in self-management interventions in CKD and normative beliefs

1. How do you think others’ feelings of self-management?

(**Probe**: government nurses, doctors, your caregivers? Other stakeholders for approving or disapproving?)

Perceived control in self-management interventions in CKD, barriers and facilitators

1. Do you feel that you would be able to manage the disease by yourself?

(**Probe**: Do you have confidence in self-management in CKD? Facilitators? Barriers?

1. How do you perceive your role in self-management?

(**Probe**: do you think you, as a patient, should be actively involved in managing your disease? Is it your role/ duty as a patient?)

**E.** Topic lists of needs toward CKD self-management

Current care

1. How do you manage CKD?

(**Probe**: What kind of support do you get of CKD care? Who, what?)

Feelings, challenges, barriers

1. How do you feel of your CKD care or the current care provided to you?

(**Probe**: What are good aspects of CKD care? What are the problems, challenges of CKD care? What are the problems, challenges of managing the CKD by yourself?)

Needs of CKD self-management

1. Is there anything else that should be done to support you to manage CKD?

(**Probe**: What for CKD management should be done in the future, when, and from whom? What support should be done to support your self-management of CKD?

**F.** Topic lists of beliefs, perceptions and needs toward eHealth

eHealth use

1. Do you know about eHealth? Have you ever used eHealth services in health care?
2. **If yes**, what was your experience in CKD? What do you think of it?

(**Probe**: motivation? How? Who offered? Who pays for it? When? Benefits? Main risks? advantages/disadvantages?)

**If not**, have you known any services for eHealth? What do you think of it?

(**Probe**: how? Who offered? When? Benefits? Main risks? advantages/disadvantages?)

Needs toward eHealth

1. What do you want of the eHealth in your health care? (**Probe**: Do you think that eHealth has an important value? Which kind of eHealth do you like or not, why?)

**Appendix 2. TOPIC LIST of focus group discussion: Patients with chronic kidney disease**

Duration Interview: 45-60 min

**A.** Introduction, explanation, consent

**B.** Demographic data:

- Identification number, sex, age, education, profession (e.g., type of work, daily routine)
- Personal and cultural background (e.g., family situation – and size, composition, religion)
- External health-related conditions (e.g., distance to a health care facility), disease stage, comorbidities

**C**. Topic lists of beliefs, perceptions and needs toward Medical Dashboard intervention

1. What is the first thing that comes to mind when you view the material?
2. How do you feel about Medical Dashboard?

(**Probe**: Do you think that it has important value? What are the advantages or disadvantages?)

1. What do you think if it is used in your clinical care?

(**Probe**: What content should be changed and how, why? What benefits do you expect from this program? What are the facilitators or barriers? Who should pay for it?)

1. Any other advice and requirement?

**Appendix 3. OBSERVATION LIST**

**Study Setting**

**Location of consultation/Clinic**

**Details** outpatient clinic/ daily clinical care

…………………………………………………………………..

(please specify)

**Healthcare worker description** Male / female Age: _____ Education: _____________________

Profession:_____________________________________________
 Total years work experience: ______________________________
 Years of work experience in community:_____________________

**Patient description** Male / female Age: Stage:
 Comorbidity: _________________________________________

**Activity**

**Observer**

**Start : AM/PM**

**End : AM/PM**

**Please circle the option that you observe. If you observe option 2, this would look like:**

**option 1 / option 2 / option 3**

**If more than one option is observed, please circle every applicable option**

**1) Belief, perception toward disease**

1. Care providers: 1. feelings of CKD 2. concerns of CKD
2. Patients: 1. Feelings of CKD 2. Changes in life 3. Impacts of CKD 4. Coping with CKD

**2) Belief, perception toward self-management**

1. Care providers: 1. Attitude 2. Subjective norms 3. Perceived control 4. Professional role
2. Patients: 1. Attitude 2. Subjective norms 3. Perceived control 4. Professional role

**3) Describe: interaction between the healthcare professional and the patient**

1. Description of the communication (e.g., adherence, future concerns, sensitive and personal issues)
2. Are there times when it has been hard for patients to follow what has been told by care providers?; Why?
3. Whether patients can ask questions openly with your doctor?

**4) Needs of CKD self-management**

**a)** Care providers: 1. Current care 2. Challenges and barriers 3. Needs

**b)** Patients: 1. Ongoing care 2. Challenges, barriers, feelings 3. Needs

**c)** Caregivers: 1. Current involvement 2. Challenges and barriers 3. Needs

**Additional remarks and observations**
