# Supplementary material for: Development and evaluation of an eHealth self-management intervention for patients with chronic kidney disease in China: protocol for a mixed-method hybrid type 2 trial
Source: BMC Nephrol. 2020 Nov 19;21:495. doi: 10.1186/s12882-020-02160-6 (PMC7678219; doi:10.1186/s12882-020-02160-6)
Supplement: Supplementary file 5 — Additional file 5. The operationalization of outcomes and descriptions of the measurement tools used in RCT. [file 12882_2020_2160_MOESM5_ESM.docx]

**Additional file 5. The operationalization of outcomes and descriptions of the measurement tools used in RCT**

| **Outcome indicator** | **Description of outcome indicators** | **Measurement tools** |
| --- | --- | --- |
|  |  |  |
| Self-management behaviour | An individual’s ability to manage the symptoms, treatment, physical and psychosocial consequences, and lifestyle changes inherent to the life with a chronic condition [1] | **Chronic Kidney Disease-Self-Management (CKD-SM) instrument**: The CKD-SM with 29 items uses a four-point Likert scale ranging from 1 (never) to 4 (always) to measure subjects' self-management behavior. Possible scores range from 29 to 116 points, with higher scores indicating better self-management behavior. Factor analysis identified four factors named self-integration, problem-solving, seeking social support and adherence to recommended regimen, accounting for 60.51% of the total variance; Cronbach's alpha coefficients for the four subscales ranged from 0.77 to 0.92. The test-retest correlation for the CKD-SM was 0.72. [2, 3] |
| Biomedical status | CKD related biomarkers | **Blood pressure**: average blood pressure after randomisation;  **Bodyweight**: average body weight after randomisation;  **Glomerular filtration rate, Serum albumin, Length, Serum calcium, Serum phosphate, Serum hemoglobin, Sodium and protein in 24h urine, albumin/creatinine ratio, Cholesterol, High-density lipoprotein, Low-density lipoprotein, Triglycerides, Hemoglobin A1C, Complications**: the records when patients visit the hospital for data collection. |
| Self-efficacy | The confidence to undertake the management of chronic disease [4] | **Chronic Kidney Disease self-efficacy (CKD-SE) scale**: The CKD-SE is a 25-item self-administered scale that measures subjects’ confidence. Responses range from no confidence (0) to the highest degree of confidence (10); possible scores range from 0 to 250 points, with higher scores indicating greater levels of confidence. Factor analysis identified four factors labelled autonomy, self-integration, problem-solving and seeking social support, accounting for 64.35% of the total variance; the internal consistency (Cronbach's alpha coefficients) of the four subscales ranged from 0.84 to 0.90, The test-retest correlation for the CKD-SE was 0.72. [3, 5] |
| Illness perception | The cognitive representations or beliefs that CKD patients have about illnesses and medical conditions | **Brief Illness perception questionnaire (BIPQ):** BIPQ has eight new items plus part of the causal scale previously used in the [Illness Perceptions Questionnaire](https://link.springer.com/10.1007%2F978-1-4419-1005-9_461) (IPQ-R). All of the items except the causal question are rated using a 0-to-10 response scale. Five of the items assess cognitive illness representations: consequences (Item 1), timeline (Item 2), personal control (Item 3), treatment control (Item 4), and identity (Item 5). Two of the items assess emotional representations: concern (Item 6) and emotions (Item 8). One item assesses illness comprehensibility (Item 7). Assessment of the causal representation is by an open-ended response item adapted from the IPQ-R, which asks patients to list the three most important causal factors in their illness (Item 9). Responses to the causal item can be grouped into categories such as stress, lifestyle, hereditary, etc., determined by the particular illness studied, and categorical analysis can then be performed. [6, 7] |
| Quality of life | An individual's perception of their position in life in the context of the culture and value systems in which they live and in relation to their goals, expectations, standards and concerns [8] | **The Kidney Disease Quality of Life 36-item short-form survey (KDQOL-36)**: The KDQOL-36 has five scales, including two generic Health-related quality of life (HRQOL) scales from the SF-12 version 1 (12 items total) and three kidney-specific scales (24 items total). The Short Form (SF-12) Physical Component Summary and Mental Component Summary are scored on a T-score metric (mean=50, SD=10, in the United States general population), with higher scores indicating better HRQOL. [9-11] |
| Mental health | The anxiety and depression in CKD patients with physical health problems | **Hospital Anxiety and Depression Scale (HADS**): The HADS was originally developed by Zigmond and Snaith (1983) [[12]](https://en.wikipedia.org/wiki/Hospital_Anxiety_and_Depression_Scale#cite_note-1) and is commonly used by doctors to determine the levels of [anxiety](https://en.wikipedia.org/wiki/Anxiety) and [depression](https://en.wikipedia.org/wiki/Depression_(mood)) that a person is experiencing. The HADS is a fourteen item scale that generates [ordinal data](https://en.wikipedia.org/wiki/Level_of_measurement). Seven of the items relate to anxiety and seven relate to depression. Zigmond and Snaith created this outcome measure specifically to avoid reliance on aspects of these conditions that are also common somatic symptoms of illness, for example, [fatigue](https://en.wikipedia.org/wiki/Fatigue_(medical)) and [insomnia](https://en.wikipedia.org/wiki/Insomnia) or [hypersomnia](https://en.wikipedia.org/wiki/Hypersomnia). It would create a tool for the detection of anxiety and depression in people with physical health problems. |
| Hospital admission | The hospital admission with an exacerbation of CKD or death due to CKD | The time to first acute hospital admission with an exacerbation of CKD or death due to CKD within nine months after randomisation |
| Health care utilisation | The quantification or description of the use of services by persons for the purpose of preventing and curing health problems, promoting maintenance of health and well-being, or obtaining information about one's health status and prognosis. | Number of hospitalisations and emergency room visits of patients, primary (community hospital or clinics) and secondary care visits |
| Cost-benefit analysis | The degree to which something is effective or productive in relation to its cost. | All costs delivering the interventions (e.g., materials used in the interventions); Medical cost (e.g., cost of treatment, hospitalization rates minored as monetary terms) |

**References**

1. Lorig KR, Holman H. Self-management education: History, definition, outcomes, and mechanisms. Ann Behav Med. 2003; 26:1-7.
2. Lin CC, Wu CC, Wu LM, Chen HM, Chang SC. Psychometric evaluation of a new instrument to measure disease self-management of the early stage chronic kidney disease patients. J Clin Nurs. 2013;22:1073-9.
3. Lin CC, Tsai FM, Lin HS, Hwang SJ, Chen HC. Effects of a self-management program on patients with early-stage chronic kidney disease: A pilot study. Appl Nurs Res. 2013;26:151-6.
4. Bandura A. Self-efficacy: Toward a unifying theory of behavioral change. Psychological Review. 1977; 84:191-215.
5. Lin CC, Wu CC, Anderson RM, Chang CS, Chang SC, Hwang SJ, et al. The chronic kidney disease self-efficacy (CKD-SE) instrument: development and psychometric evaluation. Nephrol Dial Transpl. 2012;27:3828-34.
6. Broadbent E, Petrie KJ, Main J, Weinman J. The Brief Illness Perception Questionnaire. Journal of Psychosomatic Research. 2006;60:631-7.
7. Broadbent E, Wilkes C, Koschwanez H, Weinman J, Norton S, Petrie KJ. A systematic review and meta-analysis of the Brief Illness Perception Questionnaire. Psychol Health. 2015;30:1361-85.
8. The Who Group. The World Health Organization quality of life assessment (WHOQOL): Position paper from the World Health Organization. Soc Sci Med. 1995;41: 1403-9
9. Gorodetskaya I, Zenios S, McCulloch CE, Bostrom A, Hsu CY, Bindman AB, et al. Health-related quality of life and estimates of utility in chronic kidney disease. Kidney Int. 2005;68:2801-8.
10. Cohen DE, Lee A, Sibbel S, Benner D, Brunelli SM, Tentori F. Use of the KDQOL-36 for assessment of health-related quality of life among dialysis patients in the United States. Bmc Nephrol. 2019;20:112
11. Chow SK, Tam BM. Is the kidney disease quality of life-36 (KDQOL-36) a valid instrument for Chinese dialysis patients? Bmc Nephrol. 2014;15:199.
12. Zigmond AS, Snaith RP. The hospital anxiety and depression scale. Acta Psychiatr Scand. 1983;67:361-70.
